# Charge-Potential Model of Ligand Field in Lanthanide Complexes in the Single-Electron Space

**DOI:** 10.1021/acs.inorgchem.5c00687

**Published:** 2025-04-10

**Authors:** Oliver Waldmann

**Affiliations:** Physikalisches Institut, Universität Freiburg, D-79104 Freiburg, Germany

## Abstract

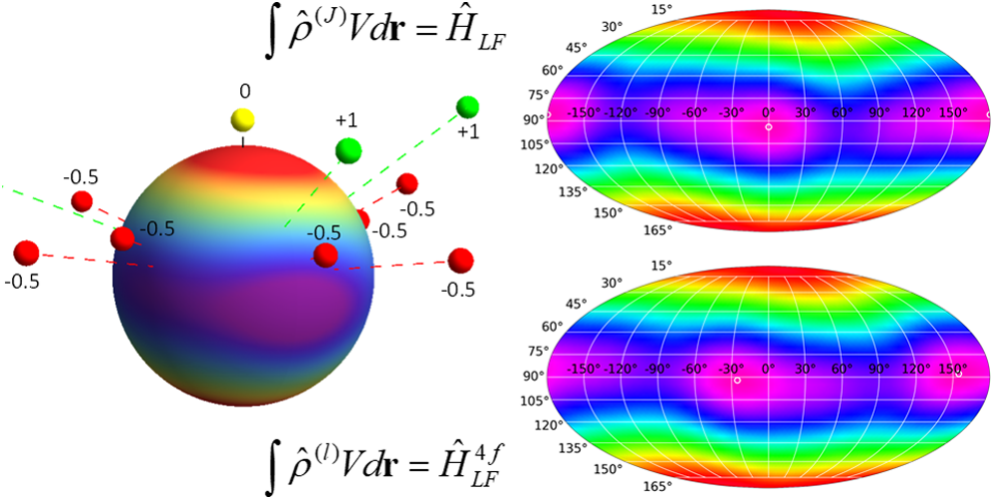

The ligand field interactions in lanthanide-based magnetic molecular complexes are crucial for their magnetic properties, and simple models for rationalizing the ligand field effects are much desired. In this work, a charge-potential model is formulated in detail, which describes the ligand field interactions as an electrostatic interaction between a generalized single-electron charge density representing the 4*f* electrons and an electrostatic potential representing the ligands. The model is equivalent to a quantum mechanical effective spin Hamiltonian in the space of the electron *f* orbitals. Furthermore, the relation with the generalized many-electron charge density and familiar effective spin Hamiltonian in the space of the ground *J* multiplet is discussed. This permits us to translate the results of any model of the ligand field splittings in the *J* multiplet, which includes high-level ab initio techniques, into the single-electron domain, and vice versa. Models based on *f* orbitals are often well suited for rationalization, and it can be hoped that the results in this work will help us better understand the effects of ligand field in lanthanide complexes.

## Abstract 2

For lanthanide complexes it is shown that the ligand field interactions can generally be understood in terms of an interaction between a generalized single-electron charge density representing the *f* orbitals and an electrostatic potential representing the ligands, as alternative to the common effective spin Hamiltonian approach. Models based on *f* orbitals often allow for an easier rationalization of the ligand field interactions, and can help us to better understand the effects of ligand field.

## Introduction

In the recent two decades magnetic molecular complexes incorporating trivalent lanthanide ions have seen renewed interest due to their fascinating magnetic properties at the molecular scale, such as single molecule magnetism (SMM) or single molecule toroidics (SMT), as well as their potential for applications in areas such as data storage and quantum information technologies. The field has comprehensively been reviewed.^1−15^ In these clusters, the magnetic properties of the lanthanide ions are governed by their ligand environments, which in a first approximation can be described by an electrostatic potential *V*(**r**) with which the electrons in the 4*f*^*n*^ shell interact through their charges, giving rise to subtle energy splittings and wave function compositions. These result in typically large magnetic anisotropies,^16^ which, in addition to the large total angular momenta *J* in the ground state as given by Hund’s rules, are the main magnetic characteristics of lanthanide ions and key to the magnetic phenomena and applications mentioned before.

The basic theories for describing the effects of ligand field interactions have been established in the last century. A box of (phenomenological) tools such as the point charge model (PCM), superposition model or angular overlap model (AOM) and their many variations and extensions has been developed^17−30^ and has more recently been complemented by ab initio methods such as the complete active space self-consistent field (CASSCF) techniques.^31−38^ The ligand field effects, when concerned with the magnetic properties, are commonly expressed in terms of an effective spin Hamiltonian (the ligand field Hamiltonian), which operates in the ground *J* multiplet, and a set of parameters (the ligand field parameters) entering this Hamiltonian.^16^ A common formulation uses the Stevens operators *Ô*_*kq*_,^39^ and the ligand field Hamiltonian reads *Ĥ*_*LF*_ = ∑_*kq*_*B*_*kq*_*Ô*_*kq*_, where the *B*_*kq*_ are the ligand field parameters (*k* and *q* will be defined below).

The ligand field Hamiltonian provides an extremely powerful tool for both describing experimental data and condensing theoretical results,^16,39^ but is often less useful for rationalizing the ligand field effects. In many lanthanide-based complexes of contemporary interest, the lanthanide ligand environments are of low symmetry or any symmetry is absent and a large number of ligand field parameters *B*_*kq*_ can be required for accurate characterization. However, the relationship between the details of the ligand environment, the ligand field parameters and the magnetic properties is highly nonlinear and complex, hampering rationalization. Ligand field models which directly consider the Coulomb interaction *W* = ∫ρ(**r**)*V*(**r**)*d***r** between the electrostatic potential *V*(**r**) representing the ligands and the charge density ρ(**r**) associated with the electrons in the 4*f*^*n*^ shell are often more intuitive. Recent prominent examples of such “charge density interacts with electrostatic potential” models (charge-potential models in brief) are the concepts discussed in ref (40), where the electron 4*f*^*n*^ charge density is characterized as oblate or prolate, or the “simple electrostatic model” (SEM) introduced in ref (41) for determining the orientation of the main magnetic anisotropy axis in Dy^III^ complexes by minimizing the electrostatic energy of the 4*f*^*n*^ charge density in the electrostatic potential. These concepts have inspired and found further application.^5,27,42,43^

Recently, a generalized 4*f*^*n*^ charge density ρ_*MM*′_^(*J*)^(**r**) has been introduced for representing the charge cloud of the 4*f*^*n*^ electrons in the *J* multiplet space.^44^ It has been shown that, for quite general conditions, the description of the ligand field splittings of the *J* multiplet in terms of the associated charge-potential model or in terms of the ligand field Hamiltonian *Ĥ*_*LF*_ are equivalent. The two pictures are thus two sides of the same coin and the equivalence allows us to seamlessly switch between the two pictures for discussion and rationalization. Representing electrons in the *J* multiplet, the generalized 4*f*^*n*^ charge density ρ_*MM*′_^(*J*)^(**r**) is inherently a many-electron object.

The ligand field effects can however also be discussed by considering the Coulomb interactions between the ligands and the lanthanide’s single-electron *f* orbitals, which is often the approach taken in textbooks to introduce ligand field theory. This suggests that a single-electron charge-potential picture, with the same potential *V*(**r**) as before but with ρ(**r**) representing the charge density of a single *f* electron, may even be more intuitive. Indeed, few recent works have taken this standpoint and found that investigating the energies of the *f* orbitals can provide substantial insight into ligand field interactions and magnetic anisotropy.^45−47^ The interesting question thus arises whether the concept of the charge-potential model, in the sense of ref (44) of an equivalence mapping connecting charge-potential and effective spin Hamiltonian domains, can also reasonably be formulated in the single-electron space.

In this work, a generalized single-electron charge density ρ_*mm*′_^(*l*)^(**r**) will be introduced such that the associated charge-potential model is equivalent to the potential matrix (*rlm*|*V*|*rlm*′) in the single-electron space (or single-electron ligand field Hamiltonian *Ĥ*_*LF*_^4*f*^), and the relationship with the *J* multiplet space will be established. Many of the individual pieces in this theory are well-known and appeared decades before in various contexts, and have recently been recollected partially and applied to lanthanide complexes of contemporary interest.^45−47^ This work contributes in several ways. The pieces will be combined into a consistent picture, which is in close analogy to the formulation in the 4*f*^*n*^ many-electron space. This rounds up the charge-potential model as a general concept and will provide deeper insight into the respective advantages of working in either of the two spaces, including a critical evaluation of the merits of the single-electron picture. Deeper insight into the significance of the generalized 4*f*^*n*^ charge density ρ_*MM*′_^(*J*)^(**r**) introduced in ref (44) will also be obtained by comparing to the generalized single-electron charge density ρ_*mm*′_^(*l*)^(**r**) introduced here. The relationship between the single-electron and *J* multiplet spaces will be interpreted as a unique mapping, which enables us to translate the results of any ligand field theory for the *J* multiplet space into the single-electron space, and vice versa, and which is a conceptual extension as compared to previous standpoints. As examples for illustration, the SEM of ref (41) will be extended to the single-electron space, and the ligand field interactions in the molecule Ln^III^DOTA^42,43,46,48−50^ discussed.

## Basics

In this section, basic and well established relations are compiled in order to provide the background and introduce the notation used in this work.

### Electrostatic Energy

The electrostatic interaction energy *W* of a charge distribution ρ(**r**) in an electrostatic potential *V*(**r**) is given by the integral^51^
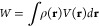
1

The electrostatic potential *V*(**r**) representing the ligand environment can be expanded in spherical harmonics,^51^ according to
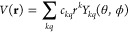
2
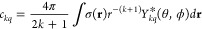
3where *Y*_*kq*_(θ, ϕ) is the *q*th component of the spherical harmonic of order *k* (*q* = −*k*, ..., *k*), θ, ϕ are the polar and azimuth angles, *r* is the radial distance, and σ(**r**) models the ligand charge density. For SI units eqs 1 and 3 should be multiplied with 1/(4π*ε*_0_) (*ε*_0_ is the dielectric constant). The potential *V*(**r**) is real, and the (complex valued) expansion coefficients thus satisfy *c*_kq_^*^ = (−1)^*q*^*c*_*k*,–*q*_.

The charge density ρ(**r**) representing the lanthanide electrons can also be expanded in spherical harmonics, yielding
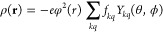
4with expansion coefficients *f*_*kq*_. They obey *f*_kq_^*^ = (−1)^*q*^*f*_*k*,–*q*_ as the charge density is also real. −*e* is the charge of an electron. The function φ(*r*) represents the radial dependence of the 4*f* single-electron wave functions in the 4*f*^*n*^ shell. The radial average is ⟨*r*^*k*^⟩ = ∫φ^2^(*r*)*r*^*k*+2^*dr*. With both *V*(**r**) and ρ(**r**) expanded in spherical harmonics, the electrostatic interaction energy becomes
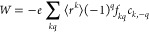
5

An immediate but important implication of eq 5 is that only contributions in the potential and charge density which are of the same order *k* can interact with each other (contributions with different *k* drop out due to the orthogonality of spherical harmonics).^44^

It is often more convenient, especially for practical applications, to switch to expansions in terms of the tesseral harmonics *Z*_*kq*_(θ, ϕ), which are related to the spherical harmonics in the standard way.^17^ For the potential one obtains
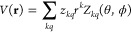
6where the expansion coefficients *z*_*kq*_ are then real valued.

In these equations *k* runs in principle through any integer with *k* ≥ 0. However, only the contributions with even *k* are relevant to the ligand field interactions due to time reversal symmetry.^16^ The *k* = 0 components correspond to constants which are irrelevant to the ligand field splittings, and thus can be omitted (in this work *k* = 0 terms shall generally be omitted).

In the PCM, the contribution of a charge *q*_*i*_ with spherical coordinates (*R*_*i*_, θ_*i*_, ϕ_*i*_) to the charge density σ(**r**) or potential *V*(**r**), respectively, is according to eq 3 given as
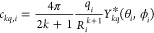
7or *z*_*kq*,*i*_ = 4π/(2*k* + 1)*q*_*i*_*R*_*i*_^–(*k*+1)^*Z*_*kq*_(θ_*i*_, ϕ_*i*_) when expressed in terms of tesseral harmonics. When multiple charges are present, their contributions obviously need to be summed up, *c*_*kq*_ = ∑_*i*_*c*_*kq*,*i*_ and similar for *z*_*kq*_.

### Effective Spin Operators

Three types of operators acting on angular momentum states will be used, namely the operators *Ŷ*_*kq*_, which transform like the spherical harmonics *Y*_*kq*_(θ, ϕ),^51,52^ the operators *Ẑ*_*kq*_, which are related to the tesseral harmonics *Z*_*kq*_(θ, ϕ),^17^ and the Stevens operators *Ô*_*kq*_.^16,39^ The angular momentum spaces of relevance are the *J* multiplet space and the single-electron space with angular momentum *l* (*l* = 3 for lanthanides). The standard basis states will be denoted as |*JM*⟩ and |*lm*⟩, with *M* = −*J*, ..., *J* and *m* = −*l*, ..., *l*, respectively.

The operators *Ŷ*_*kq*_ are defined via their matrix elements. With exploiting the Wigner–Eckart theorem,^52^ in the *J* multiplet space they are given as
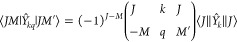
8with the reduced matrix element^53,54^
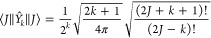
9The symbols (:::) represent Wigner 3j symbols.^52^ The matrices ⟨*JM*|*Ŷ*_*kq*_|*JM*′⟩ are thus real valued, and symmetric for *q* = 0 (else they are not symmetric). For their transpose holds ⟨*JM*′|*Ŷ*_*kq*_|*JM*⟩ = (−1)^*q*^ ⟨*JM*|*Ŷ*_*k*,–*q*_|*JM*′⟩, and they obey time reversal symmetry ⟨*J*, −*M*|*Ŷ*_*kq*_|*J*, −*M*′⟩ = (−1)^*q*^ ⟨*JM*′|*Ŷ*_*kq*_|*JM*⟩. In the *l* space, the matrix elements ⟨*lm*|*Ŷ*_*kq*_|*lm*′⟩ of *Ŷ*_*kq*_ are as in eqs 8 and 9, but with *J* and *M* replaced by *l* and *m*, respectively. They accordingly exhibit the same properties as the ⟨*JM*|*Ŷ*_*kq*_|*JM*′⟩ matrices.

The matrix elements of *Ẑ*_*kq*_, in both the *J* and *l* spaces, are derived from those of *Ŷ*_*kq*_ using the standard relationships between tesseral and spherical harmonics.^17^ The matrices ⟨*JM*|*Ẑ*_*kq*_|*JM*′⟩ and ⟨*lm*|*Ẑ*_*kq*_|*lm*′⟩ are thus real-valued and symmetric for *q* ≥ 0, and imaginary and Hermitian for *q* < 0. The Stevens operators *Ô*_*kq*_ are related to the tesseral operators as *Ẑ*_*kq*_ = *a*_*kq*_*Ô*_*kq*_, where the factors *a*_*kq*_ account for the irregular normalization of the Stevens operators. For *q* = 0 they are , , and ;^17^ a complete table of *a*_*kq*_ values is provided in Table S1. The operators *Ŷ*_*kq*_ are related to the Wybourne operators *Ĉ*_*kq*_ as .^18,55^

The *Ŷ*_*kq*_ operators will sometimes be written as *Ŷ*_*kq*_(*J*) or *Ŷ*_*kq*_(*l*), to indicate the space in which they operate. Similar convention will be applied to the operators *Ẑ*_*kq*_ and *Ô*_*kq*_ when needed. The equations in this work which are given in terms of *Y*_*kq*_ and/or *Ŷ*_*kq*_ can easily be converted into expressions with *Z*_*kq*_ and *Ẑ*_*kq*_ by replacing both *Y*_*kq*_ and *Y*_kq_^*^ with *Z*_*kq*_, and similarly so for the operators. The equations can then be easily converted to Stevens operators using *Ẑ*_*kq*_ = *a*_*kq*_*Ô*_*kq*_. For *q* = 0 the relations are *Y*_*k*0_*(θ, ϕ) = *Y*_*k*0_(θ, ϕ) = *Z*_*k*0_(θ, ϕ) and *Ŷ*_*k*0_ = *Ẑ*_*k*0_ = *a*_*k*0_*Ô*_*k*0_. In the theoretical sections mostly the *Y*_*kq*_, *Ŷ*_*kq*_ representation is used whereas in the application sections *Z*_*kq*_ and *Ô*_*kq*_ are often preferred. For all these effective spin operators, *k* can be restricted to the dimension of the considered angular momentum space.^16^ That is, it holds *k* ≤ 2*J* or *k* ≤ 2*l*, depending on the space.

In addition to these operators, the integrals (*lm*|*Y*_*kq*_|*lm*′) will be of relevance (also known as Gaunt coefficients), where (**r**|*lm*) = *Y*_*lm*_(θ, ϕ) denotes the angular part of the single-electron wave functions in position space. The standard expression^52^ is conveniently written as
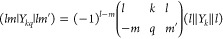
10with the reduced matrix element
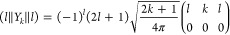
11The (*lm*|*Y*_*kq*_|*lm*′) matrices can thus be expressed in terms of *Ŷ*_*kq*_(*l*) operators as
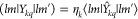
12with the factors
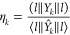
13The factors η_*k*_ are the Stevens factors for the single-electron space; values of η_*k*_ are found in, e.g., refs (16,39), and are reproduced for *f* electrons in Table 1. *k* is restricted to *k* = 2, 4, 6 for *l* = 3 by the Wigner 3j symbols (the *k* = 0 contribution is omitted). The factors η_*k*_ play a similar role in the single-electron space as the Stevens factors θ_*k*_ in the *J* multiplet space. The (*lm*|*Y*_*kq*_|*lm*′) matrices exhibit the same properties as the ⟨*lm*|*Ŷ*_*kq*_|*lm*′⟩ matrices described before.

**Table 1:** Coefficients *η*_*k*_ for *k* = 2, 4, 6 as Defined in eq 13 for Lanthanides (*l* = 3)^39^

η_2_	η_4_	η_6_
		

### Ligand Field Hamiltonian

The ligand field Hamiltonian *Ĥ*_*LF*_ in the *J* multiplet space can be written as expansion in any of the operators introduced before. For instance, when using the *Ŷ*_*kq*_(*J*) operators, it reads
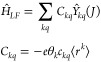
14where also the relation of the *C*_*kq*_ with the expansion coefficients *c*_*kq*_ of the potential *V*(**r**) in eq 2 is given, as it is derived in a standard first-order perturbation theory treatment.^16,17^ When expanded in terms of Stevens operators, which is the practical use case, *Ĥ*_*LF*_ reads
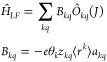
15

The coefficients *C*_*kq*_ and *B*_*kq*_ will be referred to as ligand field parameters. The values of *k* are restricted to even values and by *k* ≤ 2*J*. Terms with *k* ≥ 8 can thus in general occur. In this work only the contributions with *k* = 2, 4, 6 are considered (the range is *k* = 2, 4 when *J* ≤ 5/2, but this will be considered to be understood and not further addressed; the *k* = 0 contribution is omitted). The implications thereof will be discussed below.

### Generalized 4*f*^*n*^ Charge Density

A generalized 4*f*^*n*^ charge density ρ_*MM*′_^(*J*)^(**r**) has been introduced^44^ by requiring that the ligand field Hamiltonian in the *J* multiplet space and the electrostatic energy describing the ligand field interactions in the |*nLSJM*⟩ space be equivalent operators,
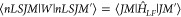
16where the |*nLSJM*⟩ are the ionic many-electron wave functions in the Russel-Saunders coupling,^56^ with the usual meaning of the symbols. The relation between *Ĥ*_*LF*_ and the generalized 4*f*^*n*^ charge density ρ_*MM*′_^(*J*)^(**r**) is then determined as
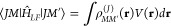
17or when expressed in operator form as
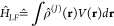
18Explicit and practically useful expressions of ρ_*MM*′_^(*J*)^(**r**) have been given as
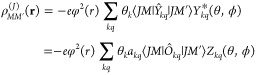
19In these equations the sum over *k* is limited to *k* = 2, 4, 6 (the *k* = 0 contribution is omitted).

The diagonal elements of the generalized 4*f*^*n*^ charge density, ρ_*MM*_^(*J*)^(**r**) (*M*′ = *M*), are the Sievers 4*f*^*n*^ charge densities.^57^ For more details on the properties of ρ_*MM*′_^(*J*)^(**r**) it is referred to ref (44).

### Potential Matrix and Single-Electron Ligand Field Hamiltonian

The single-electron wave functions in position space are written as
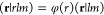
20where φ(*r*) and (**r**|*lm*) represent the radial and orbital dependencies as introduced before. The single-electron potential matrix (*rlm*|*V*|*rlm*′), or potential matrix in short, is then given as
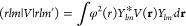
21The potential matrix is at the heart of many of the widely used (phenomenological) ligand field models, such as PCMs and AOMs.

It can be calculated from the expansion of the potential *V*(**r**), eq 2, and the addition theorem for spherical harmonics to
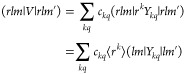
22It is occasionally more convenient to define , and write eq 22 as
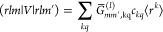
23Using orthogonality relations of the Wigner 3j symbols, eq 22 can be inverted, yielding
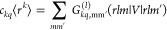
24
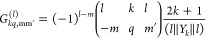
25This provides a practical equation for calculating the ligand field parameters *C*_*kq*_ (or *B*_*kq*_) from a given potential matrix (*rlm*|*V*|*rlm*′).^21^

In the above, and generally in this work, the potential matrix and expansion coefficients are expressed in terms of an electrostatic potential. However, alternative parametrizations are often more useful. For instance, AOMs parametrize the angular part of the potential matrix,^20−22,29,30^ and radial factors do not appear. The equations and results in this work can equally be applied to these types of theories by simply considering the products *c*_*kq*_⟨*r*^*k*^⟩ as model parameters.

The potential matrix can also be conveniently formulated in terms of an expansion in effective spin operators, like it is common for *Ĥ*_*LF*_.^16,45^ It is then more useful to consider the operator *Ĥ*_*LF*_^4*f*^ defined as ⟨*lm*|*Ĥ*_*LF*_^4*f*^|*lm*′⟩ = −*e*(*rlm*|*V*|*rlm*′), which will be denoted as ligand field Hamiltonian in the single-electron space. Substituting (*lm*|*V*|*lm*′) in eq 22 using eq 12 yields
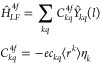
26

The *C*_*kq*_^4*f*^ (or *B*_*kq*_^4*f*^) will be denoted as ligand field parameters in the single-electron space. The close similarity to the ligand field Hamiltonian *Ĥ*_*LF*_ in eq 14 is noteworthy (although it is by construction). Some crucial differences are to be noted however. First, *Ĥ*_*LF*_^4*f*^ is, like *Ĥ*_*LF*_, an effective spin operator and thus can exactly represent the electrostatic interactions in the single-electron space. However, in *Ĥ*_*LF*_^4*f*^, *k* is strictly limited to *k* = 2, 4, 6 (the *k* = 0 contribution is omitted) by time-reversal symmetry and *k* ≤ 2 *l*, in contrast to *Ĥ*_*LF*_ where *k* ≥ 8 can occur. The implications will be discussed below. Second, the matrix of *Ĥ*_*LF*_^4*f*^ is Hermitian and exhibits time reversal symmetry, but since *l* is integer, Kramers’ degeneracy is not observed. The spectrum of *Ĥ*_*LF*_^4*f*^ thus consists in general of 2*l* + 1 distinct eigen values (except in special cases such as uniaxial ligand fields). This point will become relevant later. Lastly, the factors η_*k*_ occur instead of the Stevens factors θ_*k*_, but the coefficients *c*_*kq*_ representing the potential *V*(**r**) are the same (by construction). Therefore,
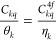
27and similarly for *B*_*kq*_ and *B*_*kq*_^4*f*^. This simple equation neatly expresses the relationship of the ligand field splittings in the *l* and *J* spaces.^16,45^

The coefficients η_*k*_ and θ_*k*_ are not equal or simply related and in fact can even be of opposite sign. The sign of η_*k*_ is given by (−1)^*k*/2^. η_2_ and η_6_ are thus negative and η_4_ is positive (Table 1). They are also independent of the lanthanide ion, in contrast to the Stevens factors θ_*k*_. Therefore, similar ligand field situations for different lanthanide complexes lead to similar ligand field parameters in the single-electron space. This is well-known, but is nevertheless a crucial feature of working in the single-electron domain, as it provides a means to meaningfully compare the ligand field interactions across different compounds, with corresponding potential for rationalization.

### Ligand Field Potential

Equations 14 and 15 allow us to construct a potential *V*_*LF*_(**r**) from any given set of ligand field parameters *B*_*kq*_ (or *C*_*kq*_),
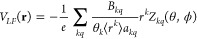
28such that the 4*f*^*n*^ charge-potential model eq 17 is strictly equivalent to the ligand field Hamiltonian *Ĥ*_*LF*_ in the *J* multiplet space.^44^ As discussed in ref (44), the potential *V*_*LF*_(**r**) is in general not identical to a potential *V*(**r**) as used for instance in a PCM, due to the holohedrization effect.^58,59^ It however—by construction—exactly includes the terms which are relevant for the ligand field splittings and thus can equally be used without restriction.

Which potential to use, *V*_*LF*_(**r**) or *V*(**r**), depends on the application. If it is applied in a first-order perturbation theory context such as in PCMs or basic AOMs, then the potential *V*(**r**) or the potential matrix as representation of it can be used. In this application the theory has “predictive power” in the sense that it permits deriving the ligand field parameters from the given potential or potential matrix. The theory will “filter out” the irrelevant components in *V*(**r**) and only those also contained in *V*_*LF*_(**r**) will determine the ligand field splittings. Otherwise, the potential *V*_*LF*_(**r**) is to be employed, and the charge-potential model establishes an equivalent operator description, which can be employed to describe the results of higher-level theories such as advanced AOMs or ab initio techniques in fact.

These statements apply to the charge-potential models in both the *J* multiplet and single-electron spaces (the latter will be introduced in the next section).^44^

## Results and Discussion

### Charge-Potential Model in the Single-Electron Space

#### Generalized Single-Electron Charge Density

The potential matrix can be formulated as charge-potential model by introducing a generalized single-electron charge density ρ_*mm*′_^(*l*)^(**r**) and requiring
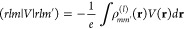
29The superscript (*l*) indicates that the generalized charge density is expressed in the 4*f* single-electron space (a superscript (*J*) indicates that it is expressed in the *J* multiplet or 4*f*^*n*^ many-electron space). Equation 21 immediately implies
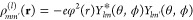
30Using the addition theorem for spherical harmonics one obtains
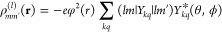
31Exploiting eq 12 one finally can write
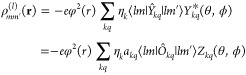
32The sum runs here over *k* = 0, 2, 4, 6 due to the properties of the Wigner 3j symbols which appear in (*lm*|*Y*_*kq*_|*lm*′). However, as in the other expressions involving a sum over *k*, the *k* = 0 term or trace shall be omitted, as it only adds a constant.

In terms of the ligand field Hamiltonian in the single-electron space, *Ĥ*_*LF*_^4*f*^, the charge-potential model, eq 29, becomes
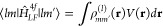
33or in operator form,
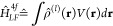
34The similarity of these results, eqs 32–34, with those for ρ_*MM*′_^(*J*)^(**r**), eqs 17–19, is satisfying.

It is noted in passing that concepts developed in ref (44) for the generalized 4*f*^*n*^ charge density ρ_*MM*′_^(*J*)^(**r**) can also be introduced for the generalized single-electron charge density ρ_*mm*′_^(*l*)^(**r**), such as eigen charge densities or spherical charge densities. These objects will however not be used in this work, and are thus not explicitly defined here for brevity. Their definitions are obvious from ref (44).

#### Significance of the Generalized Charge Densities

The generalized single-electron charge density ρ_*mm*′_^(*l*)^(**r**) is of very simple structure. The diagonal elements ρ_*mm*_^(*l*)^(**r**) (*m*′ = *m*) are according to eq 30 given as
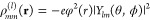
35That is, except for the radial dependence, the ρ_*mm*_^(*l*)^(**r**) are simply the squared single-electron orbitals, as it is in fact expected.

More generally, the square of any wave function (**r**|*rlμ*) = ∑_*m*_*U*_*μm*_^(l)^(**r**|*rlm*) in the single-electron space represents a physical charge density in the sense that it describes the electrostatic interactions, as given by eq 1, or eq 29 (*U*^(*l*)^ is a unitary matrix of dimension 2*l* + 1). This expresses the simple fact that, in the single-electron space, the squares of the basis functions or any linear combinations thereof represent physical spatial probability distributions. This is not true in the *J* multiplet space. Therefore, given its simple structure, in the single-electron space the notion of a generalized charge density is arguably not absolutely necessary for discussing the ligand field splittings in terms of an electrostatic interaction and might be considered dispensable. In the *J* multiplet space, however, it is a key concept.

#### Relation Between Single-Electron and Many-Electron Charge Densities

The potential *V*(**r**) appears in both the single-electron and many-electron charge-potential models. Therefore, a relation exists between ρ_*mm*′_^(*l*)^(**r**) and ρ_*MM*′_^(*J*)^(**r**), which is obtained as follows. By combining previous relations one finds
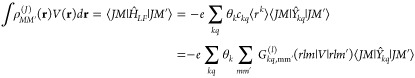
36Substituting (*rlm*|*V*|*rlm*′) using eq 29 yields
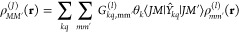
37 and sum rules results in
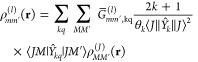
38It is not difficult to show that if ρ_*mm*′_^(*l*)^(**r**) is traceless then ρ_*MM*′_^(*J*)^(**r**) is also traceless, and vice versa. Therefore, in these equations the *k* = 0 contribution can also be omitted.

By integrating eq 37 or 38 over the potential, and exploiting eqs 17 and 33, useful relations between the matrix elements of the ligand field Hamiltonian in the single-electron and *J* multiplet spaces, ⟨*lm*|*Ĥ*_*LF*_^4*f*^|*lm*′⟩ and ⟨*JM*|*Ĥ*_*LF*_|*JM*′⟩, are obtained (the equations are not written out here for brevity since they are straightforwardly obtained as described).

It is noted that all coefficients are constants, and do not depend on the particular ligand field environment (but depend through *J* and θ_*k*_ on the type of lanthanide ion). The equations thus provide a well-defined, unambiguous, linear one-to-one mapping from the *J* multiplet (many-electron) to the 4*f* (single-electron) space, and vice versa. One can seemingly switch from one representation to the other, as both are equally valid representations of the ligand field interactions (for *k* ≤ 6, vide infra).

### Uniaxial Ligand Field

For a first illustration of the concepts, the example of an uniaxial ligand field corresponding to expansion coefficients *c*_*kq*_ = *c*_*k*0_δ_*q*,0_ is discussed. This case is well explored and understood, and allows us to compare the above methods to the previously established findings.^25,40,45−47,60^

At first, *c*_40_ = *c*_60_ = 0 will in addition be assumed (only *c*_20_ is nonzero). Although very simplistic, this scenario directly connects to the discussion of the ligand field interactions in terms of oblate or prolate 4*f*^*n*^ charge distributions of free lanthanide ions, as advocated in ref (40). The notion of oblate or prolate lanthanides is in fact bound to the sign of the Stevens factor θ_2_: θ_2_ < 0 for oblate and θ_2_ > 0 for prolate lanthanides (note that the notion cannot be taken literally since the charge density of a free ion is strictly spherical, it really just reflects θ_2_). It is thus associated with the *k* = 2 contributions in the charge density, and implicitly also to the *k* = 2 contributions in the potential (since only contributions of same order *k* interact). The *k* = 4, 6 contributions and details brought about by them are neglected. This simple scenario can nevertheless give valuable general insight into generic cases.^25,40,45^ The more general case of an uniaxial field with *c*_40_ ≠ 0 and *c*_60_ ≠ 0 will also be briefly discussed.

#### Energy Levels

For the said uniaxial ligand field, the ligand field Hamiltonian *Ĥ*_*LF*_ reduces to *Ĥ*_*LF*_ = −*ec*_20_⟨*r*^2^⟩θ_2_*Ŷ*_20_(*J*), or
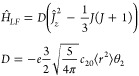
39where a common parametrization in terms of a *D* term is used (*D* = 3*B*_20_). Similarly, the 4*f* ligand field Hamiltonian becomes
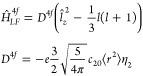
40As mentioned before, the coefficient η_2_ is negative, whereas the sign of θ_2_ varies across the lanthanides.

In order to progress further, the sign of *c*_20_ needs to be associated with the ligand environment. Let us first consider two point charges *q*_1_ = *q*_2_ = −*e* located on the *z* axis at positions ±*R*. This mimics ligand environments with an axial elongation along the *z* axis. From eq 3 one directly infers *c*_*kq*_ = −*e*4π/(2*k*+1)*R*^–(*k*+1)^ [*Y*_kq_^*^(0, 0) + *Y*_kq_^*^(π, 0)] or *c*_20_ = *Q*4π/5*R*^–3^*Y*_20_(0, 0), with *Q* = *q*_1_ + *q*_2_ = −2*e*. Since *Y*_20_(0, 0) > 0, *c*_20_ is negative in this ligand environment (*Q* < 0 is assumed). Positive values of *c*_20_ are obtained for ligand environments where the ligand charge density is concentrated near the equator, i.e., close to the *xy* plane. A simple model could be a square planar arrangement of four point charges (each of charge −*e*) located in the *xy* plane at positions (*x*, *y*) = (±*R*, ±*R*). Equation 3 then yields *c*_*kq*_ = −*e*4π/(2*k*+1)*R*^–(*k*+1)^ ∑_*n* = 0_^3^*Y*_kq_^*^(90°, 45° + *n*90°), or *c*_20_ = *Q*4π/5*R*^–3^*Y*_20_(90°, 0), with *Q* = −4*e*. Since *Y*_20_(90°, 0) < 0, *c*_20_ is positive in this ligand environment. The essence of these models is more generally valid. When the square of four charges is located above or below the *xy* plane, or when two such squares are placed above and below in a symmetric sandwich-like configuration, one obtains for the *q* = 0 contributions
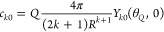
41or *c*_20_ = *Q*4π/5*R*^–3^*Y*_20_(θ_*Q*_, 0) ∝ (3 cos^2^θ_*Q*_ – 1). Here, θ_*Q*_ is the polar angle of the charges, and *Q* is given by the sum of the charges representing the ligand(s). This result is also obtained when each “sheet” consists of 3 or 5 or more charges on a ring, with *Q* appropriately adapted. Essentially, when the ligand environment is reasonably well approximated by one or two “sheets” of negative charge above and/or below the *xy* plane or close to the equator, *c*_20_ is negative when θ_*Q*_ < 54.7°, and positive when θ_*Q*_ > 54.7° (54.7° is the magic angle, cos^2^ θ = 1/3).

For the axially elongated ligand environment or *c*_20_ < 0, respectively, *D*^4*f*^ is thus negative, see eq 40. Therefore, the single-electron *f* orbitals |*l*, ±*l*) are ground state, whereas the *f* orbital |*l*, 0) is most destabilized. This fully aligns with the textbook expectation that in this ligand environment the *f*_*z*^3^_ orbital should experience the strongest Coulomb repulsion as it is the most prolate *f* orbital and its main lobes point directly toward the negative ligand charges. The orbitals *f*_*x*(*x*^2^–3*y*^2^)_ and *f*_*y*(3*x*^2^–*y*^2^)_ in contrast are the most oblate *f* orbitals and thus should be lowest in energy by this argument. The situation is obviously reversed for the equatorial ligand environment (*c*_20_ > 0), where *D*^4*f*^ > 0. Here, the *f*_*z*^3^_ orbital should experience the least Coulomb repulsion and be ground state as its main lobes nicely point away from the ligand charges in the equatorial plane. Figure S1 sketches these situations.

For discussing the ligand field splittings in the *J* multiplet one needs to specify the lanthanide ion. Let us consider Ce^III^ first. Here, θ_2_ is negative, corresponding to an oblate 4*f*^*n*^ charge distribution.^40,60^ Accordingly, by considering the Coulomb interaction, it is expected that in the axially elongated ligand environment (*c*_20_ < 0) the states where the oblate’s axis points along the *z* axis exhibit the least repulsion and are ground state. That is, the states |*J*, ±*J*⟩ are predicted to be ground state. This nicely correlates with eq 39, which for the given situation yields *D* < 0 and indeed a |*J*, ±*J*⟩ ground state. For the equatorial ligand environment (*c*_20_ > 0) the situation reverses, as then the oblate’s axis should point maximally away from the *z* axis and lie in the *xy* plane for achieving a low energy. This again matches with *D* > 0, which implies that the minimally polarized states |*J*, ±1/2⟩ or |*J*, 0⟩ are ground state, depending on whether *J* is half-integer or integer, respectively. These considerations obviously carry over to those lanthanides with oblate 4*f*^*n*^ charge distribution or θ_2_ < 0, respectively. For prolate lanthanides with θ_2_ > 0, the lowest and highest energy configurations are reversed as compared to the θ_2_ < 0 case. Here the prolate’s axis wants to stay away from the ligand charges for achieving a minimal energy configuration.

These scenarios in the single-electron and *J* multiplet spaces are summarized in Table 2. The well-known conclusions^25,40,45,60^ are reproduced.

**Table 2:** Ground States in the 4*f* Single-Electron (2nd Row) and *J* Multiplet (3rd and 4th Rows) Spaces for the Uniaxial Ligand Field Environments Discussed in the Text^a^

	*c*_20_ < 0 (axially elongated)	*c*_20_ > 0 (equatorial)
η_2_ < 0	*D*_4*f*_ < 0	*D*_4*f*_ > 0
	|*l*, ±*l*⟩	|*l*, 0⟩
	*f*_*x*^3^–3*x*y^2^_, *f*_3*x*^2^y–*y*^3^_	*f*_*z*^3^_
θ_2_ < 0	*D* < 0	*D* > 0
(oblate)	|*J*, ±*J*⟩	|*J*, 0⟩,
θ_2_ > 0	*D* > 0	*D* < 0
(prolate)	|*J*,0⟩,	|*J*, ±*J*⟩

a θ_2_ < 0 and *θ*_2_ > 0 correspond to oblate and prolate 4*f*^*n*^ charge distributions, respectively.

When the higher order terms *c*_40_ and *c*_60_ are nonzero, a case by case analysis for each lanthanide ion is essentially needed.^45,47^ The following general result is possible, however. The energy spectra in the single-electron and *J* multiplet spaces are connected. For instance, integrating eq 37 and noting that (for the uniaxial ligand field) the diagonal elements of the Hamiltonian matrices are the energy eigenvalues *ε*_*M*_ and *ε*_*m*_^4*f*^, respectively, one obtains a simple linear relationship for these energies. When expressed in terms of Stevens operators, it reads
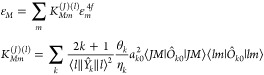
42A similar equation relating the *ε*_*m*_^4*f*^ to the *ε*_*M*_ can be found along the same lines. The prefactors *K*_*Mm*_^(*J*)(*l*)^ generated from eq 42 are given for the example of Dy^III^ in Table 3, and for other trivalent lanthanides in Table S2. In the tables it has been taken into account that the levels for *m* ≠ 0 are doubly degenerate. For instance, for the Dy^III^ ion’s *M* = ±15/2 and *M* = ±1/2 levels one finds *ε*_±15/2_ = 0.429 *ε*_3_^4*f*^ + 0.429 *ε*_2_^4*f*^ – 0.571 *ε*_1_^4*f*^ – 0.286 *ε*_0_^4*f*^ and *ε*_±1/2_ = −0.453 *ε*_3_^4*f*^ + 0.177 *ε*_2_^4*f*^ + 0.366 *ε*_1_^4*f*^ – 0.090 *ε*_0_^4*f*^, respectively. The prefactors do not match those given in refs (45,47), since the latter are not trace-preserving while those from eq 42 are (in this work the trace, or center of gravity, is chosen to be zero). However, the energy splittings determined by eq 42 and from refs (45,47) are exactly identical. For instance, the energy difference is *ε*_±15/2_ – *ε*_±1/2_ = 0.881 *ε*_3_^4*f*^ + 0.252 *ε*_2_^4*f*^ – 0.937 *ε*_1_^4*f*^ – 0.196 *ε*_0_^4*f*^.

**Table 3:** Factors *K*_*Mm*_^(*J*)(*l*)^ of Eq 42 for Dy^III^^a^

*M*	*m* = 3	*m* = 2	*m* = 1	*m* = 0
±15/2	1287	1287	–1716	–858
±13/2	1287	–715	286	–858
±11/2	858	–1001	143	0
±9/2	264	–627	–297	660
±7/2	–330	–143	–319	792
±5/2	–828	227	109	492
±3/2	–1179	441	696	42
±1/2	–1359	531	1098	–270

a The degeneracy of the *f* orbitals for *m* = ±3, ±2, ±1 is taken into account by doubling the respective values of *K*_*Mm*_^(*J*)(*l*)^. All values in the table need to be divided by 3003.

The classification suggested in ref (47) of the (trivalent) lanthanide ions into the subgroups oblate-01 (Ce, Tb), oblate-1 (Pr, Dy), oblate-02 (Nd, Ho), prolate-31 (Er, Pm), and prolate-3 (Sm, Tm, Yb) simply groups the lanthanide ions according to the signs of their Stevens factors θ_4_ and θ_6_ (oblate-01 = +–, oblate-1 = −+, oblate-02 = −–, prolate-31 = ++, and prolate-3 else). Since the θ_4_, θ_6_ signs are equal for lanthanides with *f*^*n*^ and *f*^*n*+7^, the θ_4_, θ_6_ sign pattern appears to also naturally explain the “*f*^*n*+7^ effect”.^46^

As a summary of this subsection it can be concluded that previously known results emerge naturally and straightforwardly as special cases from the theory.

#### Generalized Charge Density

In the previous subsection the effects of the Coulomb interactions were discussed by resorting to the canonical *f* orbitals, but obviously the squares of the *f* orbitals were considered, as these represent charge distributions, and not the wave functions. Some comments are in place.

In a uniaxial ligand field the energy eigen states in the *l* space are the states |*lm*⟩ themselves, and the diagonal elements of the generalized charge density ρ_*mm*_^(*l*)^(**r**) are then the correct charge densities to be considered in the above discussion (in the terminology of the charge-potential concept,^44^ the ρ_*mm*_^(*l*)^(**r**) then represent the eigen charge densities in the *l* space). This statement is true also in the general situation of a uniaxial ligand field with *c*_40_ ≠ 0 and *c*_60_ ≠ 0. However, as discussed in the context of eq 35, the ρ_*mm*_^(*l*)^(**r**) are essentially just the squared *f* orbitals, and the discussion falls back to using those. That is, the discussion in the previous subsection happened to be correct. It follows, however, that if the ligand field is not uniaxial (or not very close to it), then it is not correct to discuss the ligand field interactions in terms of the squared canonical single-electron *f* orbitals. This is the critical essence of eqs 29, 33, 34.

Another point to consider is that the interactions between electrostatic potential and charge density are per *k* order, as expressed in eq 5. For the uniaxial ligand field where only *c*_20_ is nonzero it follows that *W* = −*e*⟨*r*^2^⟩*c*_20_*f*_20_. That is, only the contribution ρ_00_^(*l*)(*k* = 2)^(**r**) ∝ – *eφ*^2^(*r*)*Y*_20_(θ, ϕ) in the charge density is of relevance (when one writes ρ_*mm*_^(*l*)^(**r**) = ∑_*k*_ρ_*mm*_^(*l*)(*k*)^(**r**), with obvious meaning of the ρ_*mm*_^(*l*)(*k*)^(**r**)). It is in this case then more meaningful to describe the *f* electron in terms of a squared *d* orbital instead of a squared *f* orbital. If *c*_40_ and *c*_60_ are also nonzero, then all the *k* = 2, 4, 6 contributions of the charge density participate in the electrostatic interaction, but the significance of each is given by *f*_*kq*_*c*_kq_^*^ and not by *f*_*kq*_. This is a further important essence of charge-potential models.^44^

When the ligand field is not uniaxial or not otherwise simple in nature, the relation between the energy spectrum and eigen functions in the single-electron space and those in the *J* multiplet space becomes in general complicated, and will not reduce to simple results such as eq 42. This is the essence of eqs 37 and 38. When interested in the magnetic properties and other observables related to the *J* multiplet, working in the single-electron space can then become less beneficial.

### Simple Electrostatic Model in the Single-Electron Space

In the SEM introduced in ref (41) the electrostatic interaction energy *W*, eq 1, is calculated using the Sievers 4*f*^*n*^ charge density ρ_*JJ*_^(*J*)^(**r**) for the maximally polarized state |*J*, ±*J*⟩, with the charge density however rotated into a direction specified by polar and azimuthal angles β and α. This rotated charge density can be written as ρ_SEM_^(*J*)^(β, α, **r**) = *R̂*(*αβ*)ρ_*JJ*_^(*J*)^(**r**), where *R̂*(*αβ*) is the rotation operator associated to the angles α, β (a third Euler angle γ can be omitted as it only induces irrelevant phases). The potential *V*(**r**) is suggested to be obtained from a PCM with the ligand charges given by the formal Lewis charges. The interaction energy then becomes a functional *W*_SEM_(β, α) = ∫ρ_*SEM*_^(*J*)^(β, α, **r**)*V*(**r**)*d***r**, which is minimized with respect to these angles. The minimizing angles are proposed to correspond to the direction of the main magnetic anisotropy axis. This model can also be formulated in terms of the ligand field Hamiltonian *Ĥ*_*LF*_, where it then relates to rotating the states |*J*, ±*J*⟩ by the angles β and α, and minimizing the expectation value *E*(β, α) = ⟨*JJ*|*R̂*^†^(*αβ*)*Ĥ*_*LF*_*R̂*(*αβ*)|*JJ*⟩.^44^

The conditions for the applicability of the SEM are well established.^41,44^ Without going into details they can coarsely be stated as applicable (#1) to Kramers ions and (#2) when the ligand field is sufficiently uniaxial, such that the states |*J*, ±*J*⟩ approximate the ground state and are separated from excited states by a sufficiently large energy gap. In the context of this work, condition #2 means that when the ligand coordinate frame is rotated into the appropriate orientation, the coefficient *c*_20_ is dominating and is of the correct sign for stabilizing the |*J*, ±*J*⟩ states (see Table 2).

The idea underlying the SEM can be carried over to the single-electron space. The equations given in previous subsections, when expressed in terms of *Y*_*kq*_ and *Ŷ*_*kq*_, are obviously consistent with the transformation properties of the rotation group and thus can be rotated by applying the rotation operator *R̂* or the associated Wigner *D* matrices.^52^ Compared to the SEM in the *J* multiplet space, two subtleties need to be considered however. First, the single-electron ligand field Hamiltonian *Ĥ*_*LF*_^4*f*^ does not exhibit Kramers’ degeneracy, and the SEM needs to be extended accordingly. Second, although a general relationship between the Hamiltonian matrix elements in the *l* and *J* multiplet spaces exists, as discussed before, a simple general result is not available for determining the ground states and which of the |*lm*⟩ orbitals correspond to the |*J*, ±*J*⟩ states.

The latter point can be addressed to some extent by specifying that the ligand field parameters or the coefficients *c*_*kq*_, respectively, are such that the ground state in the *l* space can be ascribed to either the orbitals |*l*, ±*l*⟩ or to |*l*, 0⟩ and that it is sufficiently well separated energy-wise from the excited states. In addition, condition #2 for the ground state in the *J* multiplet space has to be fulfilled. Such a ligand field will henceforth be called “strongly uniaxial”. Table 2 then remains valid and describes the possible scenarios, if *c*_20_ is understood to refer to its value in a ligand coordinate frame in which the main magnetic anisotropy axis is (nearly) aligned along the *z* axis. In this coordinate frame, the SEM essentially boils down to the uniaxial case considered before. The SEM is accordingly applicable in the two cases of (i) an oblate lanthanide ion in an axially elongated ligand environment and (ii) a prolate lanthanide ion in an equatorial ligand environment. It is mentioned that these conditions are overly restrictive, i.e., the SEM might be applicable when the ligand field is not as strongly uniaxial as suggested here. In that case the applicability needs however to be investigated for the specific situation at hand.

Case (ii) is discussed first, as it is simpler. Here, θ_2_ > 0 and *c*_20_ > 0, and in a strongly uniaxial ligand field the single-electron orbital |*l*, 0⟩ is ground state and nondegenerate. A rotation of the |*JJ*⟩ ground state in the *J* multiplet space according to *R̂*(*αβ*)|*JJ*⟩ is equivalent to a rotation of the |*l*, 0⟩ state in the *l* space according to *R̂*(*αβ*)|*l*, 0⟩. The SEM thus consists of minimizing the expectation value *E*(β, α) = ⟨*l*, 0|*R̂*^†^(*αβ*)*Ĥ*_*LF*_^4*f*^*R̂*(*αβ*)|*l*, 0⟩. Equivalently, and more intuitively, eq 1 can be used as suggested originally,^41^ and the optimal orientation of the generalized single-electron charge density ρ_*l*_^00^(**r**) be determined, i.e., the functional *W*_SEM_(β, α) = ∫[*R̂*(*αβ*)ρ_*l*_^00^(**r**)]*V*(**r**)*d***r** be minimized. Since ρ_*l*_^00^(**r**) is essentially just the squared *f*_*z*^3^_ orbital, in this case it should indeed be very simple to rationalize the orientation of the main anisotropy axis by using the SEM in the single-electron picture.

Case (i) requires more effort. Here θ_2_ < 0 and *c*_20_ < 0, and in a strongly uniaxial ligand field the ground state is related to the single-electron orbitals |*l*, ±*l*⟩. However, Kramers’ degeneracy does not apply, and the low-energy sector is formed by two, nondegenerate eigen states with a small energy gap and wave functions consisting predominantly of a mixture of the two orbitals (these properties are implied by the condition of strongly uniaxial ligand field). The effective Hamiltonian matrix in the space of this quasi-degenerate doublet can thus be modeled as
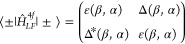
43where |±⟩ = *R̂*(*αβ*)|*l*, ±*l*⟩ and with obvious definition of the entries *ε*(β, α) and Δ(β, α). The energy eigenvalues are trivially obtained as ϵ_±_(β, α) = *ε*(β, α) ± |Δ(β, α)|. The angle dependence of |Δ(β, α)| will in general be complicated, but the maximum value of |Δ(β, α)| is expected to be small. It is thus proposed to ignore this contribution or to take the average of the two energy levels, respectively, and to consider the expectation value *E*(β, α) = *ε*(β, α) ≡ ⟨+|*Ĥ*_*LF*_^4*f*^|+⟩ in the minimization process. By following through calculations given in ref (44), this can be shown to be equivalent to minimizing *W*_SEM_(β, α) where the rotated charge density ρ_*ll*_^(*l*)^(**r**), which is essentially the squared *f*_*x*(*x*^2^–3*y*^2^)_ orbital, is used (equivalently the rotated ρ_–*l*,–*l*_^(*l*)^(**r**) or squared *f*_*y*(3*x*^2^–*y*^2^)_ orbital, respectively, can be used). A possible refinement would be to minimize *E*(β, α) = (⟨+|*Ĥ*_*LF*_^4*f*^|+⟩ + ⟨−|*Ĥ*_*LF*_^4*f*^|−⟩)/2. In this way the non-Kramers case is broken down to the same procedure as for the Kramers case.

It is mentioned in passing, that the criteria for judging the applicability of the SEM suggested in ref (44) for the *J* multiplet SEM can largely be carried over to the SEM in the single-electron space. The comparison to the ground state predicted by the ligand field Hamiltonian *Ĥ*_*LF*_^4*f*^ appears to be particularly suitable.

The connection between the SEMs in the single- and many-electron spaces can be made explicit. By expressing the effect of *R̂* on the 4*f*^*n*^ charge density ρ_*JJ*_^(*J*)^(**r**) in terms of Wigner *D* matrices, using a special result for the Wigner *D* matrices and the orthogonality of the spherical harmonics, it has been shown in ref (44) that the interaction energy *W*_SEM_ in the many-electron space can be written as
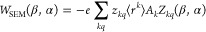
44with factors
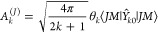
45where the superscript (*J*) indicates the *J* multiplet space (the *A*_*k*_^(*J*)^ match the factors *A*_*k*_ of ref (57)). Going through the same calculation but for the rotated single-electron charge density ρ_SEM_^(*l*)^(β, α, **r**) = *R̂*(*αβ*)ρ_*mm*_^(*l*)^(**r**) one obtains again eq 44 but with the factors
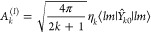
46They are listed in Table 4.

**Table 4:** Factors *A*_*k*_^(*l*)^ of Eq 46 for *f* Electrons with *m* = 0, ..., ±3 (*l* = 3)^a^

	*k* = 2	*k* = 4	*k* = 6
*m* = 0			
*m* = ±1			
*m* = ±2	0		
*m* = ±3			
*A*_*k*_^(*J*)^ (*Dy*^*III*^)			

a The last row gives the factors *A*_*k*_^(*J*)^ of eq 45 for Dy^III^ (*J* = 15/2) and M = ±15/2.^57^.

For both SEMs the interaction energy *W*_SEM_ is a sort of “weighted” electrostatic potential, i.e., essentially is the electrostatic potential but with weighted *k* contributions, where the weight factors are however different for the two SEMs. Therefore, when the *k* = 2 contributions in the ligand field are dominating, as implied by the condition of strongly uniaxial ligand field, the SEM in the single- and many-electron spaces will yield similar minimizing angles; in the limit where the *k* = 4, 6 contributions are zero they will be exactly identical. When the *k* = 4, 6 contributions become more significant or even dominant, when the two SEMs will produce increasingly different predictions (unless the direction of the main anisotropy axis is essentially determined by, e.g., symmetry, in which case the SEM is not very useful anyway).

In view of the above, using the SEM in the single-electron space instead of in the *J* multiplet space appears attractive. For the given conditions the resulting minimizing angles (and thus orientation of the main magnetic anisotropy axis) will be similar, but working with (squared) *f* orbitals instead of Sievers 4*f*^*n*^ charge densities might be more appealing. When the ligand field is not strongly uniaxial the SEM in the two spaces may give different results, and it needs to be investigated which of the two is more realistic. In any case, to conclude this subsection, a single-electron SEM can be an additional useful tool.

### Simple Electrostatic Model for Non-Kramers Ions in the *J* Multiplet Space

As a corollary of the previous subsection it is noted that the proposed method for handling the SEM in the single-electron space can obviously also provide an approach for extending the SEM in the *J* multiplet space to non-Kramers ions. Here one would define |±⟩ = *R̂*(*αβ*)|*J*, ±*J*⟩ as in ref (44), and the energy to minimize would be given by *E*(β, α) = ⟨+|*Ĥ*_*LF*_|+⟩, by the same line of reasoning as in the previous subsection. The *J* multiplet SEM has in fact been applied before to non-Kramers ions,^27^ but to the best of the authors knowledge a formal argument justifying the method was not given.

### Ln^III^DOTA

Next, lanthanide complexes with the DOTA ligand shall be looked at, where H_4_DOTA is tetraazacyclododecane—*N*, *N*′, *N*″, *N*‴—tetraacetate. These molecules have attracted interest since the eight carboxylate oxygen atoms in the DOTA ligand (only four are coordinated to the lanthanide ion) lie essentially in a plane, creating an equatorial ligand environment (*c*_20_ > 0 in the notation of this work). Initially the complexes Na[Ln^III^DOTA(H_2_O)]. 4H_2_O (or [Ln^III^DOTA(H_2_O)Na_3_]^2+^ in short) were investigated in which a water molecule is coordinated in the apical position, leading to a pseudo 4-fold symmetry axis with surprisingly strong effects on the magnetic anisotropy axes.^42,46,48−50^ Subsequently, the apical water molecule could be removed, yielding the series of isostructural [Me_4_N][Ln^III^DOTA].2Me_4_NCL·5H_2_O complexes (Ln^III^DOTA in short) with strict tetragonal symmetry, with Ln^III^ = Tb^III^, Dy^III^, Ho^III^, Er^III^, Tm^III^, and Yb^III^.^43^ For these, the experimentally observed ground states and first excited states in the *J* multiplet could be well described with a surprisingly simple model, which exploits the Sievers 4*f*^*n*^ charge densities ρ_*MM*_^(*J*)^(**r**) and their presumed interaction with the coordinating carboxylate oxygen charges located at polar angles of θ_*Q*_ ≈ 63°. In this subsection the tetragonal complexes Ln^III^DOTA will be considered; the cluster [Dy^III^DOTA(H_2_O)Na_3_]^2+^ will be touched upon in the next subsection. A detailed discussion of the ligand field interactions in these molecules is beyond the scope of this work, but the example can further illustrate the methods developed in the above.

The model proposed in ref (43) for Ln^III^DOTA assumes a strictly uniaxial ligand field ignoring tetragonal contributions, and orders the |*JM*⟩ energy levels by the magnitude of the associated Sievers 4*f*^*n*^ charge density in the direction θ = θ_*Q*_ of the carboxylate oxygen atoms: The smaller the magnitude of ρ_*MM*_^(*J*)^(θ_*Q*_) the lower the energy of the associated |*JM*⟩ level, with the argument that a lower charge density suggests a weaker electrostatic repulsion.^43^ The energies of the |*JM*⟩ levels are thus predicted to follow ϵ_*M*_ ∝ −*eρ*_*MM*_^(*J*)^(θ_*Q*_) (the azimuthal angle can be dropped for *M*′ = *M* or *q* = 0, the radial dependence is irrelevant in this model).

It has to be mentioned that this model does not comply with the laws of electrostatics and in this sense is nonphysical; the electrostatic interaction energy is not given by evaluating a charge distribution ρ(**r**) in the direction of a charge *q* but by the integral eq 1. This point, however, shall be ignored and not further discussed here. Instead, a charge-potential model will be set up such that it produces the same predictions as this model. While this may appear preposterous, it is entirely in the spirit of the concepts introduced in ref (44) and here. The key results here and there were derived assuming an electrostatic potential and first-order perturbation theory, but shown to actually provide an equivalence mapping from one domain to the other and to not be limited by these assumptions. In ref (44) the equivalence was exploited for representing the results of ab initio theory in terms of a charge-potential model, which thus represented also nonelectrostatic effects such as covalency or effects due to configuration interaction. The equivalence is not restricted to electrostatic models; it can be applied to represent any model (including nonphysical ones).

The sought-after potential *V*_*LF*_(**r**) can easily be found by requiring that eq 17 produces the same level scheme as the proposed model, i.e., by equating ϵ_*M*_ = ⟨*JM*|*Ĥ*_*LF*_|*JM*⟩∝ −*eρ*_*MM*_^(*J*)^(θ_*Q*_) ∝ ∑_*k*_θ_*k*_*a*_*k*0_⟨*JM*|*Ô*_*k*0_|*JM*⟩*Y*_*k*0_(θ_*Q*_), where eq 19 was used in the last step. The model thus corresponds to ligand field parameters *B*_*k*0_ = *ã θ*_*k*_*a*_*k*0_*Y*_*k*0_(θ_*Q*_), where the (positive) constant *ã* accounts for the unspecified energy scale. Equations 15 or 14 then directly yields *c*_*k*0_ = *ãY*_*k*0_(θ_*Q*_)/(−*e*⟨*r*^*k*^⟩). Knowing the potential, the energy levels in the single-electron space can be determined from eq 26, which results in *C*_*k*0_^4*f*^ = *ãη*_*k*_*Y*_*k*0_(θ_*Q*_), or
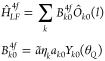
47The energy spectrum of the single-electron *f* orbitals is thus given by ϵ_*m*_^4*f*^ = ∑_*k*_*B*_*k*0_^4*f*^⟨*lm*|*Ô*_*k*0_|*lm*⟩ in this model. The *f* orbital spectra are equal for the six Ln^III^DOTA molecules, since the factors η_*k*_ are. The *J* multiplet spectra in contrast vary across the series because of the dependence of the Stevens factors θ_*k*_ on the lanthanide ion.

**Figure 1:**
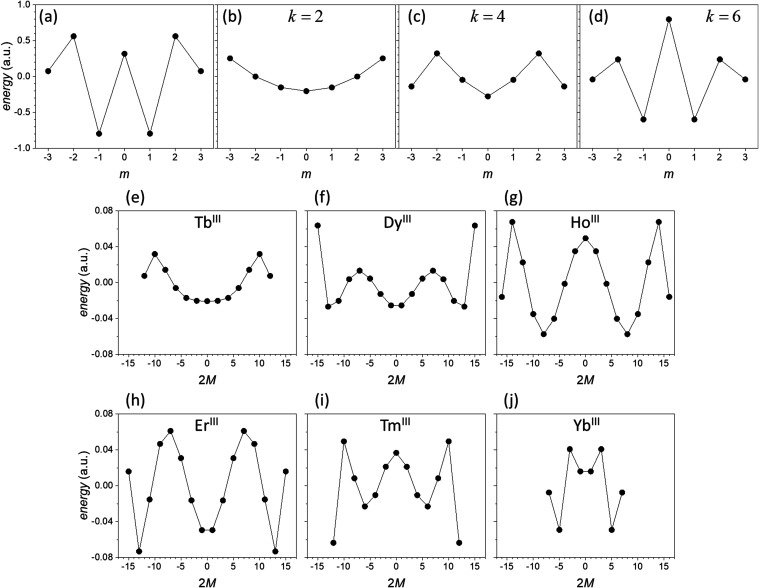
Energy spectra of Ln^III^DOTA according to the model of ref (43) (θ_*Q*_ = 63°). Panel (a) shows the energy spectrum of the *f* orbitals in the single-electron space; panels (b), (c) and (d) show the individual contributions *B*_*k*0_^4*f*^⟨*lm*|*Ô*_*k*0_|*lm*⟩ for each *k*. The *f* orbital spectrum is identical for all Ln^III^DOTA molecules. Panels (e) to (j) show the energy spectra in the *J* multiplet space for the six molecules Tb^III^DOTA to Yb^III^DOTA. These energies and the resulting ground and first excited states were discussed before in ref (43). In all panels the energies are given in arbitrary units, since the energy scale (factor *ã*, see e.g., eq 47) is undetermined in the model.

The energy spectra in the single-electron and *J* multiplet spaces, as predicted by the model of ref (43) and the above relationships, are plotted for the six Ln^III^DOTA molecules in Figure 1, as a function of the magnetic quantum number *m* or *M* as appropriate for the respective space. The energy spectra in both the *f* orbital and *J* multiplet spaces are obviously not well described by a parabolic *m*^2^ or *M*^2^ dependence, i.e., are not well approximated by the *k* = 2 contribution. Accordingly, the oblate-vs-prolate “rule” as compiled in Table 2 is not obeyed. It is in fact a characteristic feature of the model of ref (43) to produce exceptionally large higher order contributions. The *k* = 6 contribution is actually dominating in the *f* orbital space, as evident from Figure 1b–d, which present the contribution of each *k* order to the *f* orbital energies. The large higher order contributions in the single-electron space implies large higher order contributions in the *J* multiplet space, explaining the large deviations from a *M*^2^ behavior. This feature of the model is not an artifact but is corroborated by the large fourth and sixth order ligand field parameters determined in ref (43) from fits to experimental data and ab initio methods. The bottom line here is that—by translating the model of ref (43) into the single-electron space—the ground and first excited states in the series of six Ln^III^DOTA complexes can be consistently and comprehensively rationalized by the spectrum of the *f* orbitals.

The significance of the higher order *k* = 4, 6, terms with respect to the *k* = 2 contribution can be evaluated by the ratios *c*_*k*0_/*c*_20_. They are predicted in the model to vary as *c*_*k*0_/*c*_20_ = *Y*_*k*0_(θ_*Q*_)⟨*r*^2^⟩/(*Y*_20_(θ_*Q*_) ⟨*r*^*k*^⟩), which are essentially of order one. A PCM along the lines discussed before, with result eq 41, would yield ratios *c*_*k*0_/*c*_20_ = 5*Y*_*k*0_(θ_*Q*_)/[(2*k*+1)*R*^*k*–2^*Y*_20_(θ_*Q*_) ](note that due to holohedrization, a square of four charges above the lanthanide ion is equal to a sandwich of two squares of charges above and below with *Q* adapted accordingly, and that thus eq 41 applies to Ln^III^DOTA). Since *R* ∼ 2.3 Å,^43^ the trivial factor 5/(2*k*+1)*R*^–*k*+2^ drops by ∼0.11 and ∼0.014 for *k* = 4 and 6, respectively. This drop by orders of magnitude could be lessened within the PCM by displacing the charges, e.g., by bringing them closer to the lanthanide center to account for covalency, or substituting with several charges.^24,26−28^ It however appears unrealistic that the large higher order contributions suggested by the model could be accounted for by a realistic modified PCM. AOMs could be better candidates for obtaining a realistic, phenomenological modeling. They do not include such radial dependencies and tend to produce stronger higher-order terms than PCMs.

**Figure 2:**
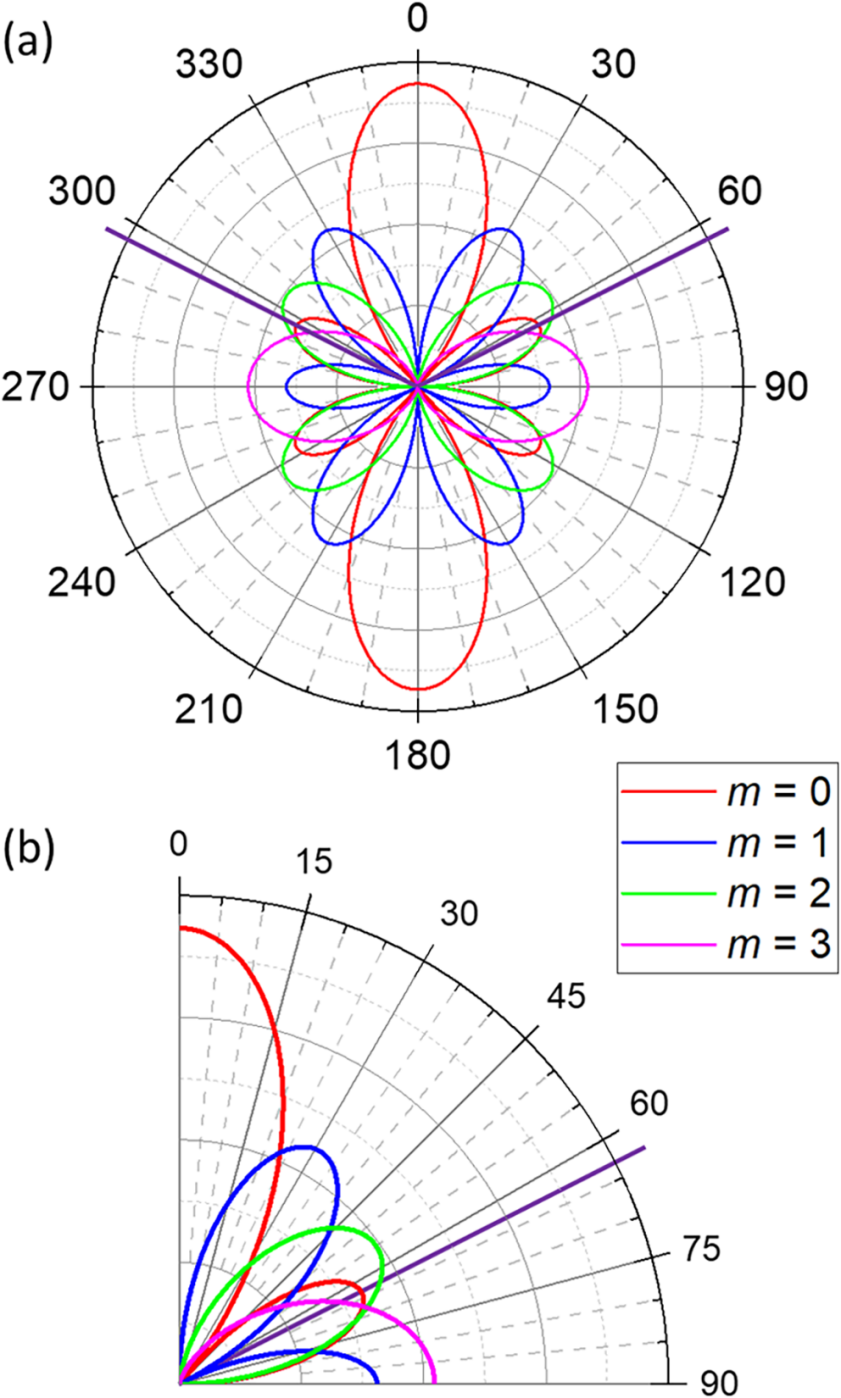
Polar plots of the magnitude of the *f* orbitals for *m* = 0 (red), *m* = 1 (blue), *m* = 2 (green), and *m* = 3 (magenta) (the polar plots for *m* < 0 are equal to those for *m* > 0). The magnitude instead of the square of the orbitals is plotted for better visibility. Panel (a) shows the polar angle range θ = 0...360°, and panel (b) provides a zoom into the range θ = 0...90°. In both panels the dark pink lines mark the direction θ_*Q*_ = ±63°, which is the direction of the coordinating carboxylate oxygen atoms in the Ln^III^DOTA molecules.

The argument underlying the model of ref (43) can also be applied to the *f* orbitals. Indeed, eq 47 implies for the *f* orbital energies that ϵ_*m*_^4*f*^ ∝ ∑_*k*_η_*k*_*a*_*k*0_⟨*lm*|*Ô*_*k*0_|*lm*⟩*Y*_*k*0_(θ_*Q*_) ∝ −*eρ*_*mm*_^(*l*)^(θ_*Q*_). Since the diagonal elements of the generalized single-electron charge density are essentially the squared *f* orbitals, eq 35, one arrives at the simple relation ϵ_*m*_^4*f*^ ∝ |*Y*_*lm*_(θ_*Q*_)|^2^. That is, in the single-electron space the sequence of *f* orbital energies follows the magnitude of the *f* orbital charge densities in the direction of the carboxylate oxygens. This immediately explains why the *m* = ±1 orbitals are ground state, with a large energy gap to the higher-lying orbitals (see Figure 1a): The *m* = ±1 orbitals exhibit a zero at cos^2^(θ) = 1/5 or θ ≈ 63.4°, which is very close to the polar angle θ_*Q*_ ≈ 63° of the oxygen atoms. The sequence of the other orbitals is less intuitive, but follows from the scheme. Figure 2 presents polar plots of |*Y*_*lm*_(θ)| (this is shown instead of |*Y*_*lm*_(θ)|^2^ for reasons of better presentation). The magnitudes of the charge densities in the direction of θ = θ_*Q*_ increase in the order *m* = ±1, ±3, 0, ±2, which of course matches the order shown in Figure 1a. Therefore, in the single-electron space, the ground state of Ln^III^DOTA is easily rationalized. Even though the model’s argument is nonphysical, it can be expected that the zero of the *m* = ±1 orbitals in the direction of the oxygen atoms will also result in a stabilization of these orbitals in more realistic models and thus could be identified as a defining feature of the ligand environment in Ln^III^DOTA.

In order to infer the ground state in the *J* multiplet space from the knowledge of the ground *f* orbital, eq 42 or Table S2 has to be worked through for each type of lanthanide ion. A simple clear-cut argument for inferring the ground state in the *J* multiplet space from the *f* orbital energies seems not to be possible. This illustrates the previous comment, that the mapping from the single-electron to the *J* multiplet space (and vice versa) can be less intuitive, even in relatively simple ligand environments, such as the uniaxial ligand field assumed in the above model.

### [Dy^III^DOTA(H_2_O)Na_3_]^2+^

Lastly, as an example of the application of the SEM in the single-electron space and comparison to the *J* multiplet SEM, the molecule [Dy^III^DOTA(H_2_O)Na_3_]^2+^ is briefly discussed (*J* = 15/2). The DOTA ligand, as discussed in the previous subsection, is expected to create an equatorial ligand environment (*c*_20_ > 0). In [Dy^III^DOTA(H_2_O)Na_3_]^2+^ the polar angle of the coordinating oxygens is θ_*Q*_ ≈ 70°, and thus even larger than in Ln^III^DOTA (and the ligand field closer to equatorial). According to Table 2, the  level should be stabilized. However, experiments indicated a maximally polarized ground state M = ±*J* with an orientation of the main magnetic anisotropy axis (nearly) perpendicular to the molecule’s pseudo C_4_ symmetry axis, which initially was ascribed to the presence of the apical water molecule in [Ln^III^DOTA(H_2_O)Na_3_]^2+^.^42,46,48−50^ A subsequent SEM analysis in the *J* multiplet space successfully reproduced the experimental finding, using a PCM for the electrostatic potential which in addition to the eight charges representing the eight carboxylate oxygen atoms in the DOTA ligand also included the three Na^+^ cations which bind to three of the four carboxylate arms (carboxylate oxygen charges = −1/2*e*, Na^+^ charges = +1*e*, the part of the potential which is of relevance for the ligand field interactions or contributes to *V*_*LF*_, respectively, is denoted as *V*_*DOTANa*_3__ in brief).^41^ In this SEM, the orientation of the main magnetic anisotropy axis was found to agree very well with ab initio results, and has been attributed to the presence of the three Na^+^ ions in this ligand sphere.

The *J* multiplet SEM assumes a ground state of  nature, which for the *V*_*DOTANa*_3__ potential is in contradiction with the earlier discussion which resulted in Table 2 and which gives a  ground level. The contradiction is not resolved by the presence of the Na^+^ charges, since due to their large distances from the Dy^III^ center their effect on the potential *V*_*DOTANa*_3__ is small, and the classification of the ligand field as equatorial remains fully intact. In fact, a conventional PCM calculation for *V*_*DOTANa*_3__ yields a ground state with a strong  nature (contribution ∼ 67%), in full agreement with Table 2. The resolution of this contradiction and explanation of the seeming success of the SEM is an interesting topic, but goes beyond the scope of this work. Here the focus will be on comparing the results of the SEM in the single-electron space to the result of the *J* multiplet SEM reported in ref (41), i.e., the SEMs using the *M* = *J* and *m* = *l* charge densities ρ_*JJ*_^(*J*)^(**r**) and ρ_*ll*_^(*l*)^(**r**), respectively.

**Figure 3:**
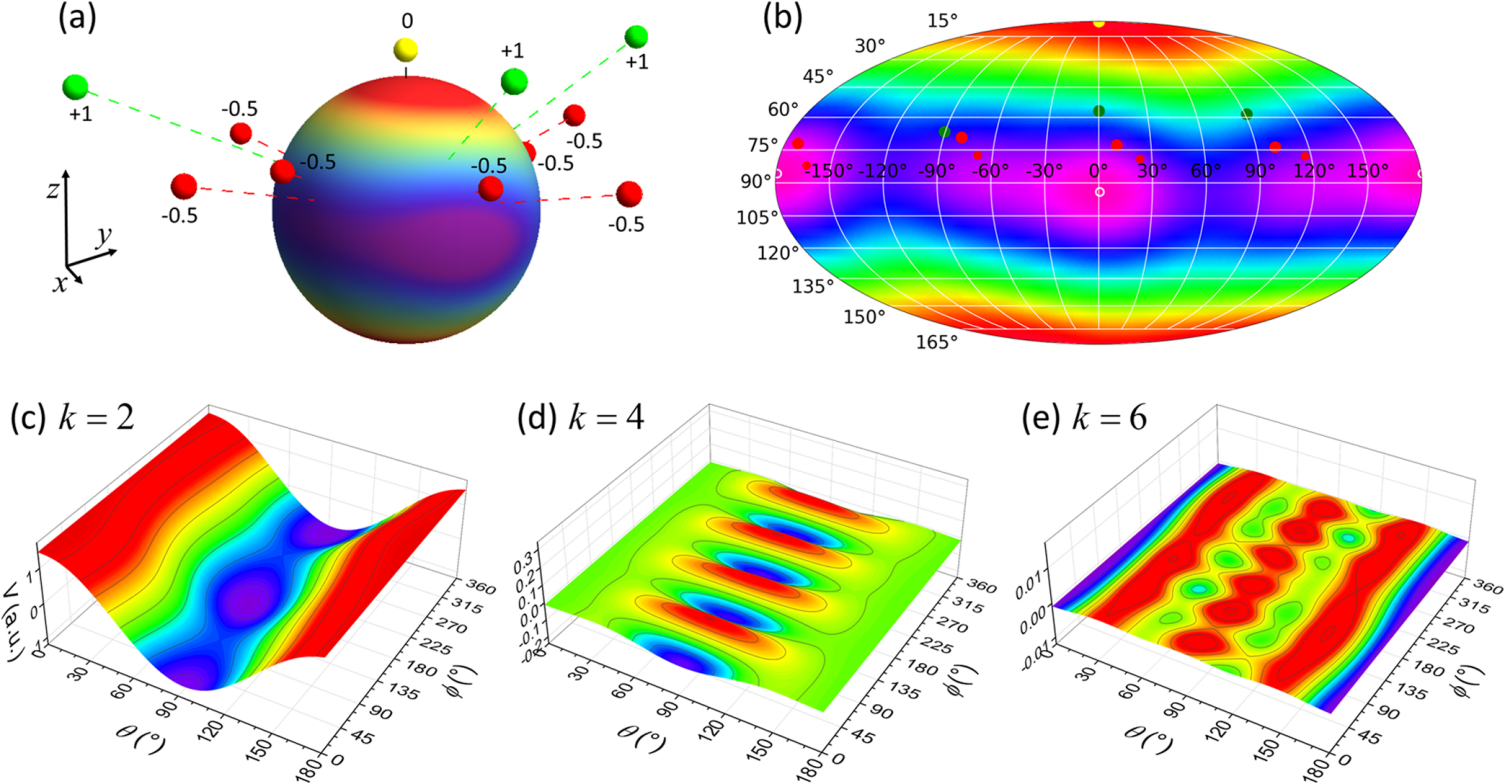
Ligand field potential *V*_*DOTANa*_3__(**r**) for the molecule [Dy^III^DOTA(H_2_O)Na_3_]^2+^ (*r* = 0.4752 Å). In the panels violet represents most negative and red most positive values, and units are arbitrary. (a) 3D surface contour plot for a side view (*z* axis pointing up). The red, yellow, and green balls represent the carboxylate oxygens (charge ), oxygen of the apical H_2_O molecule (charge 0), and Na^+^ cations (charge +*e*), respectively (the coordinates are given in Table S3). The *z* axis is in the direction of the apical oxygen and the *x* axis in the direction of the “middle” Na^+^ ion. The potential is clearly of equatorial nature, and in the equatorial plane the most negative areas (violet spots) are in the *x* direction. (b) Mollweide contour plot of *V*_*DOTANa*_3__. The red, yellow, and green circles represent the ligands similar to panel (a). The smaller red circles represent the noncoordinating carboxylate oxygens which are at larger distances. The white open circles represent the minima of *V*_*DOTANa*_3__. Panels (c–e) show 2D surface contour plots of the *k* = 2, 4, and 6 contributions to the potential, respectively. The *k* = 2 contribution is dominant. The *k* = 4 contribution is much weaker and slightly modifies the potential along the equator. The *k* = 6 contribution can be considered negligible.

The spherical dependence of the ligand field potential *V*_*DOTANa*_3__(**r**) is shown in Figure 3 in various representations. Since the potential is also dependent on the radial distance *r*, the choice of *r* for plotting needs some consideration. Due to the *r*^*k*^ radial dependence in eq 2, the *k* = 2 contribution would be emphasized for small values of *r*, whereas for large values of *r* the *k* = 6 contribution would become dominant. Choosing *r* is thus somewhat arbitrary. In this work,  Å has been used as it appears to reflect the relative importance of the *k* = 2, 4, 6 contributions reasonably well. The coordinate frame for representing the ligands has been defined such that the *z* axis points into the direction of the oxygen of the apical H_2_O molecule and the *x* axis is in the direction of the “middle” Na^+^ cation.

Figures 3a,b demonstrate the equatorial nature of the ligand field *V*_*DOTANa*_3__. The areas of negative charge (violet and blue) are clearly concentrated at polar angles of θ ≈ 90°, whereas the positive charge areas (red) are located at the poles. The Mollweide representation used in Figure 3b is described in detail in ref (61). The ligand field is dominated by the *k* = 2 contribution, with spots of negative charge in the direction of the carboxylate oxygens (Figure 3c). The *k* = 4 contribution is substantially weaker, but leads to an additional modulation along the equator (Figure 3d). The *k* = 6 contribution is negligibly small (Figure 3e).

**Figure 4:**
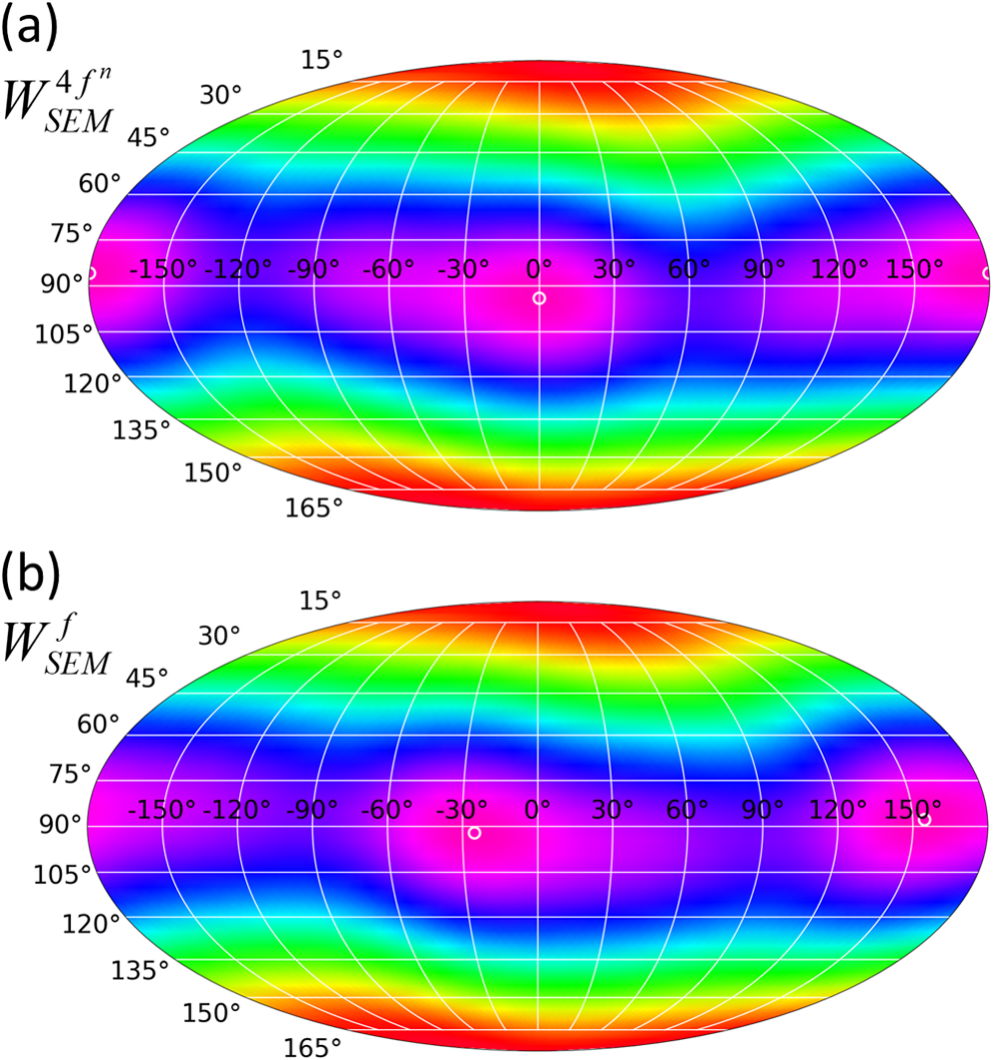
Mollweide contour plots of the SEM energy landscapes for *V*_*DOTANa*_3__ in (a) the *J* multiplet and (b) the single-electron spaces (*M* = *J* = 15/2, *m* = *l* = 3). In both panels violet represents most negative and red most positive values, and units are arbitrary. The white open circles represent the minima in the respective energy landscape.

The energy landscapes of the SEM in the *J* multiplet and single-electron spaces, *W*_SEM_^4*f*^*n*^^ = ∫*R̂ρ*_*JJ*_^(*J*)^*Vd***r** and *W*_SEM_^*f*^ = ∫*R̂ρ*_*ll*_^(*l*)^*Vd***r**, respectively, are presented in Figure 4 as Mollweide contour plots. Both landscapes are very similar to the spherical dependence of the potential *V*_*DOTANa*_3__, which is expected from the dominance of the *k* = 2 contribution in *V*_*DOTANa*_3__. In fact, as discussed before in the context of eq 44, if the *k* = 4, 6 ligand field contributions would be zero, they would be identical to each other as well as to the potential *V*_*DOTANa*_3__, except of some scaling factors. That is, in this case the SEMs simply would reduce to determining the direction of the minimal negative areas in the ligand field potential. In the case of *V*_*DOTANa*_3__ the fourth order contribution is however not negligible and leads to subtle differences in the energy landscapes along the equator. The spherical dependencies of *V*_*DOTANa*_3__ and *W*_SEM_^4*f*^*n*^^ are in fact very similar to each other. This is however a coincidence and related to the fact that for Dy^III^ the factors *A*_*k*_^(*J*)^ are of equal sign for *k* = 2, 4 (see Table 4), such that the *k* = 2 and 4 contributions add up in a similar way as in the potential *V*_*DOTANa*_3__. Accordingly, the locations of the minima are nearly identical too (*V*_*DOTANa*_3__: θ_*min*_ = 94.14°, ϕ_min_ = 0.69°; *W*_SEM_^4*f*^*n*^^: θ_min_ = 94.07°, ϕ_min_ = 0.02°; indicated by white open circles in Figures 3b and 4a). This is different for *W*_SEM_^*f*^. Here *A*_4_^(*l*)^ for *m* = 3 is of opposite sign (see Table 4), and the fourth order contribution in the potential induces shifts of the minima along the equator in the energy landscape by ∼25° (*W*_SEM_^*f*^: θ_min_= 92.14°, ϕ_min_ = −25.25°; see white open circles in Figure 4a).

Apparently, in the example of [Dy^III^DOTA(H_2_O)Na_3_]^2+^ the orientation of the main anisotropy axis as predicted by the *J* multiplet and single-electron SEMs is largely determined by the *k* = 2 contribution in the *V*_*DOTANa*_3__ ligand field potential, but the fourth order contribution, albeit weak, leads to significant differences with regards to the azimuthal angle. Given its easier rationalization, the single-electron SEM appears to be useful to get a coarse idea, but the *J* multiplet SEM appears to be more appropriate with regards to accuracy. This further illustrates the point made before, that when interested in observables which are related to the *J* multiplet space for a particular molecule, a discussion in the single-electron space, while equivalently possible as demonstrated in this work, may become less preferred.

## Conclusions

The concept of the charge-potential model has previously been introduced as an alternative description of the ligand field splittings of the *J* multiplet, which is equivalent to the common quantum mechanical effective spin Hamiltonian in the *J* multiplet space.^44^ In this work, the concept has been extended to the single-electron space or space of *f* orbitals, respectively. A generalized single-electron charge density has been introduced and the resulting charge-potential model shown to be equivalent to the potential matrix or ligand field Hamiltonian in the single-electron space, respectively. In addition, and in particular, the relations connecting the single-electron and *J* multiplet spaces have been given.

As demonstrated by means of the example of the Ln^III^DOTA complexes, the result of any model or theory which can describe the ligand field interactions in the *J* multiplet space, which includes high-level models such as advanced AOMs or in fact ab initio techniques, can be mapped into the single-electron space and represented by an electrostatic ligand field potential *V*_*LF*_(**r**) and the generalized single-electron charge density ρ_*mm*′_^(*l*)^(**r**) (or by the single-electron ligand field Hamiltonian). In this mapping, all effects including those which are not of purely electrostatic origin or not within the reach of first-order perturbation theory, such as covalency or configuration interaction, are correctly and equivalently represented in the single-electron model—as far as they manifest themselves in orders with *k* ≤ 6. Effects associated with *k* > 6 contributions in the *J* multiplet ligand field Hamiltonian cannot be represented in the single-electron space. A *k* ≤ 6 restriction has also been given for the *J* multiplet charge-potential model in ref (44). However, as indicated there, in the *J* multiplet space the charge-potential model can be formulated to overcome this constraint.^44^ This is not possible in the single-electron space, and the *k* ≤ 6 limitation is a fundamental restriction in this space. Regardless, given that *k* ≤ 6 implies that one may have to deal with up to 27 ligand field parameters, one will often restrict to sixth order for practical reasons. Also, the *k* > 6 contributions may be weak or negligible, especially for lanthanides, and ab initio and other high-level codes may not report these terms. That is, the *k* ≤ 6 limitation in the single-electron space, while fundamental, might often be of little practical relevance.

As compared to the *J* multiplet representation, a main benefit of working in the single-electron space is certainly its (near) independence on the type of lanthanide ion. This allows us to more clearly correlate the ligand field splittings (in the single-electron space) to the ligand environment. However, when one is interested in the magnetic properties and other observables which are related to the *J* multiplet, any finding needs eventually to be mapped from the single-electron into the *J* multiplet space. Although this mapping is given by mathematically simple, linear equations, it is not easily rationalized. In sufficiently simple situations, such as uniaxial ligand fields or otherwise simple ligand environments, the *f* orbital energies may relatively easily be correlated to the energies and wave functions in the *J* multiplet space. In the more general case of a lower symmetry ligand field, however, when the eigen states in the single-electron space exhibit a more complicated energy spectrum and are complicated compositions of the canonical *f* orbitals, the translation into the *J* multiplet space will be less transparent. This raises questions with regards to the general usefulness of the single-electron representation. The single-electron charge-potential model formulated in this work has been investigated for few generic cases, which however were limited to situations with strict or strongly uniaxial ligand field. This has not been by accident, but for that reason. Further efforts are therefore needed in order to reach a better understanding of the concept’s capabilities for rationalizing the ligand field splittings in lanthanide complexes. The recent examples in the literature, where the ligand field and magnetic anisotropy in lanthanide complexes have been analyzed with benefit using single-electron language,^45−47^ are certainly encouraging.

It appears that one tool may not fit it all, and that their combination may provide the greatest benefit in terms of rationalizing the ligand field splittings. The comprehensive and consistent set of techniques and methods outlined in this work in conjunction with ref (44) provides a solid and general framework to this end. It can be hoped that it will spur further insights into the matter and help us to better understand the effects of ligand field in lanthanide complexes.

Last but not least, besides the single-electron or *J* multiplet spaces, the ligand field interactions can also be discussed in the “intermediate” space of total orbital angular momentum *L*, similar as for 3*d* ions.^16,32,33,35,45,62^ With the methods and procedures established here and in ref (44), it is straightforward to also formulate the charge-potential model in the *L* space. As shown in ref (45), carefully analyzing the ligand field interactions also in the *L* space can give valuable insight into the relevance of individual mechanisms contributing to the ligand field splittings. It is far from obvious however that this space would offer benefits in terms of equivalence mappings and rationalization, given it is neither directly connected to magnetic observables nor has the benefit of direct connection to the ligand environment or independence from the lanthanide ion. Lastly, the results in this work are not limited to lanthanide complexes but also apply to transition metal ions and actinides.

## Computational Details

Numerical values given in tables were calculated using the Wolfram Mathematica program package.

## Supporting Information Available

Pictorial representation of the ground and excited states in the *f* orbital single-electron space for an uniaxial ligand field (Figure S1); factors *a*_*kq*_ for *k* = 2, 4, 6 (Table S1); factors *K*_*Mm*_^(*J*)(*l*)^ for the trivalent lanthanide ions (Table S2); coordinates and charges of the ligand atoms defining the ligand field potential *V*_*DOTANa*_3__ (Table S3) (PDF)

## References

[ref1] Woodruff D. N.; Winpenny R. E. P.; Layfield R. A. Lanthanide single-molecule magnets. Chem. Rev. 2013, 113, 5110–5148. 10.1021/cr400018q. 23550940

[ref2] Ungur L.; Lin S.-Y.; Tang J.; Chibotaru L. F. Single-molecule toroics in Ising-type lanthanide molecular clusters. Chem. Soc. Rev. 2014, 43, 6894–6905. 10.1039/C4CS00095A. 24975197

[ref3] Layfield R. A.; Murugesu M. Lanthanides and Actinides in Molecular Magnetism; Wiley, 2015.

[ref4] McAdams S. G.; Ariciu A.-M.; Kostopoulos A. K.; Walsh J. P.; Tuna F. Molecular single-ion magnets based on lanthanides and actinides: Design considerations and new advances in the context of quantum technologies. Coord. Chem. Rev. 2017, 346, 216–239. 10.1016/j.ccr.2017.03.015.

[ref5] Liu J.-L.; Chen Y.-C.; Tong M.-L. Symmetry strategies for high performance lanthanide-based single-molecule magnets. Chem. Soc. Rev. 2018, 47, 2431–2453. 10.1039/C7CS00266A. 29492482

[ref6] Long J.; Guari Y.; Ferreira R. A.; Carlos L. D.; Larionova J. Recent advances in luminescent lanthanide based Single-Molecule Magnets. Coord. Chem. Rev. 2018, 363, 57–70. 10.1016/j.ccr.2018.02.019.

[ref7] Gaita-Ariño A.; Luis F.; Hill S.; Coronado E. Molecular spins for quantum computation. Nat. Chem. 2019, 11, 301–309. 10.1038/s41557-019-0232-y. 30903036

[ref8] Aromí G.; Roubeau O. Handbook on the Physics and Chemistry of Rare Earths. In Including Actinides; Elsevier, 2019; Vol. 56, pp 1–54.

[ref9] Wernsdorfer W.; Ruben M. Synthetic Hilbert Space Engineering of Molecular Qudits: Isotopologue Chemistry. Adv. Mater. 2019, 31, e1806687 10.1002/adma.201806687. 30803060

[ref10] Moreno-Pineda E.; Wernsdorfer W. Measuring molecular magnets for quantum technologies. Nat. Rev. Phys. 2021, 3, 645–659. 10.1038/s42254-021-00340-3.

[ref11] Marin R.; Brunet G.; Murugesu M. Shining New Light on Multifunctional Lanthanide Single-Molecule Magnets. Angew. Chem., Int. Ed. 2021, 60, 1728–1746. 10.1002/anie.201910299. 31596534

[ref12] Vignesh K. R.; Rajaraman G. Strategies to Design Single-Molecule Toroics Using Triangular Ln3 n Motifs. ACS Omega 2021, 6, 32349–32364. 10.1021/acsomega.1c05310. 34901588 PMC8655769

[ref13] Bernot K. Get under the Umbrella: A Comprehensive Gateway for Researchers on Lanthanide-Based Single-Molecule Magnets. Eur. J. Inorg. Chem. 2023, 26, e202300336 10.1002/ejic.202300336.

[ref14] Wang J.; Sun C.-Y.; Zheng Q.; Wang D.-Q.; Chen Y.-T.; Ju J.-F.; Sun T.-M.; Cui Y.; Ding Y.; Tang Y.-F. Lanthanide Single-molecule Magnets: Synthetic Strategy, Structures, Properties and Recent Advances. Chem. – Asian J. 2023, 18, e202201297 10.1002/asia.202201297. 36802202

[ref15] Chiesa A.; Santini P.; Garlatti E.; Luis F.; Carretta S. Molecular nanomagnets: a viable path toward quantum information processing?. Rep. Prog. Phys. 2024, 87, 034501 10.1088/1361-6633/ad1f81. 38314645

[ref16] Abragam A.; Bleaney B. Electron Paramagnetic Resonance of Transition Ions, International series of monographs on physics; Clarendon P: Oxford, 1970.

[ref17] Hutchings M. T. Point-Charge Calculations of Energy Levels of Magnetic Ions in Crystalline Electric Fields. Solid State Phys. 1964, 16, 227–273. 10.1016/S0081-1947(08)60517-2.

[ref18] Wybourne B. G. Spectroscopic Properties of Rare Earths; John Wiley & Sons: New York, 1965.

[ref19] Newman D. J.; Ng B. The superposition model of crystal fields. Rep. Prog. Phys. 1989, 52, 699–762. 10.1088/0034-4885/52/6/002.

[ref20] Schäffer C. E.; Jørgensen C. K. The angular overlap model, an attempt to revive the ligand field approaches. Mol. Phys. 1965, 9, 401–412. 10.1080/00268976500100551.

[ref21] Urland W. On the ligand-field potential for f electrons in the angular overlap model. Chem. Phys. 1976, 14, 393–401. 10.1016/0301-0104(76)80136-X.

[ref22] Schönherr T.; Atanasov M.; Adamsky H. Comprehensive Coordination Chemistry II; Elsevier, 2003; Vol. 2, pp 443–455.

[ref23] Malta O. L. A simple overlap model in lanthanide crystal-field theory. Chem. Phys. Lett. 1982, 87, 27–29. 10.1016/0009-2614(82)83546-X.

[ref24] Porcher P.; Couto Dos Santos M.; Malta O. Relationship between phenomenological crystal field parameters and the crystal structure: The simple overlap model. Phys. Chem. Chem. Phys. 1999, 1, 397–405. 10.1039/a803807d.

[ref25] Baldoví J. J.; Cardona-Serra S.; Clemente-Juan J. M.; Coronado E.; Gaita-Ariño A.; Palii A. Rational design of single-ion magnets and spin qubits based on mononuclear lanthanoid complexes. Inorg. Chem. 2012, 51, 12565–12574. 10.1021/ic302068c. 23102271

[ref26] Baldoví J. J.; Clemente-Juan J. M.; Coronado E.; Gaita-Ariño A.; Palii A. An updated version of the computational package SIMPRE that uses the standard conventions for Stevens crystal field parameters. J. Comput. Chem. 2014, 35, 1930–1934. 10.1002/jcc.23699. 25087575

[ref27] Jiang S.-D.; Qin S.-X. Prediction of the quantized axis of rare-earth ions: the electrostatic model with displaced point charges. Inorg. Chem. Front. 2015, 2, 613–619. 10.1039/C5QI00052A.

[ref28] Dun Z.; Bai X.; Stone M. B.; Zhou H.; Mourigal M. Effective point-charge analysis of crystal fields: Application to rare-earth pyrochlores and tripod kagome magnets R3Mg2Sb3O14. Phys. Rev. Res. 2021, 3, 023012 10.1103/PhysRevResearch.3.023012.

[ref29] Bronova A.; Bredow T.; Glaum R.; Riley M. J.; Urland W. BonnMag: Computer program for ligand-field analysis of f n systems within the angular overlap model. J. Comput. Chem. 2018, 39, 176–186. 10.1002/jcc.25096. 29143342

[ref30] Suta M.; Cimpoesu F.; Urland W. The angular overlap model of ligand field theory for f elements: An intuitive approach building bridges between theory and experiment. Coord. Chem. Rev. 2021, 441, 213981 10.1016/j.ccr.2021.213981.

[ref31] Ungur L.; Chibotaru L. F. Ab Initio Crystal Field for Lanthanides. Chem.—Eur. J. 2017, 23, 3708–3718. 10.1002/chem.201605102. 27983776

[ref32] Atanasov M.; Ganyushin D.; Sivalingam K.; Neese F. Molecular Electronic Structures of Transition Metal Complexes II; Transition Metal Complexes I. I.; Mingos D. M. P.; Day P.; Dahl J. P., Eds.; Structure and Bonding; Springer Berlin Heidelberg: Berlin, Heidelberg, 2012; Vol. 143, pp 149–220.

[ref33] Singh S. K.; Eng J.; Atanasov M.; Neese F. Covalency and chemical bonding in transition metal complexes: An ab initio based ligand field perspective. Coord. Chem. Rev. 2017, 344, 2–25. 10.1016/j.ccr.2017.03.018.

[ref34] Dey S.; Sharma T.; Sarkar A.; Rajaraman G. Computational Modelling of Molecular Nanomagnets; Rajaraman G., Ed.; Challenges and Advances in Computational Chemistry and Physics; Springer International Publishing: Cham, 2023; Vol. 34, pp 291–394.

[ref35] Jung J.; Atanasov M.; Neese F. Ab Initio Ligand-Field Theory Analysis and Covalency Trends in Actinide and Lanthanide Free Ions and Octahedral Complexes. Inorg. Chem. 2017, 56, 8802–8816. 10.1021/acs.inorgchem.7b00642. 28708410

[ref36] Jung J.; Islam M. A.; Pecoraro V. L.; Mallah T.; Berthon C.; Bolvin H. Derivation of Lanthanide Series Crystal Field Parameters From First Principles. Chem.—Eur. J. 2019, 25, 15112–15122. 10.1002/chem.201903141. 31496013

[ref37] Gupta T.; Singh M. K.; Rajaraman G. Organometallic Magnets; Chandrasekhar V.; Pointillart F., Eds.; Topics in Organometallic Chemistry; Springer International Publishing: Cham, 2019; Vol. 64, pp 281–354.

[ref38] Soncini A.; Piccardo M. Ab initio non-covalent crystal field theory for lanthanide complexes: a multiconfigurational non-orthogonal group function approach. Phys. Chem. Chem. Phys. 2022, 24, 18915–18930. 10.1039/D1CP05488K. 35913488

[ref39] Stevens K. W. H. Matrix Elements and Operator Equivalents Connected with the Magnetic Properties of Rare Earth Ions. Proc. Phys. Soc., Sect. A 1952, 65, 209–215. 10.1088/0370-1298/65/3/308.

[ref40] Rinehart J. D.; Long J. R. Exploiting single-ion anisotropy in the design of f-element single-molecule magnets. Chem. Sci. 2011, 2, 2078 10.1039/c1sc00513h.

[ref41] Chilton N. F.; Collison D.; McInnes E. J. L.; Winpenny R. E. P.; Soncini A. An electrostatic model for the determination of magnetic anisotropy in dysprosium complexes. Nat. Commun. 2013, 4, 2551 10.1038/ncomms3551. 24096593

[ref42] Boulon M.-E.; Cucinotta G.; Luzon J.; Degl’Innocenti C.; Perfetti M.; Bernot K.; Calvez G.; Caneschi A.; Sessoli R. Magnetic anisotropy and spin-parity effect along the series of lanthanide complexes with DOTA. Angew. Chem., Int. Ed. 2013, 52, 350–354. 10.1002/anie.201205938. 23208792

[ref43] Manvell A. S.; Pfleger R.; Bonde N. A.; Briganti M.; Mattei C. A.; Nannestad T. B.; Weihe H.; Powell A. K.; Ollivier J.; Bendix J.; Perfetti M. LnDOTA puppeteering: removing the water molecule and imposing tetragonal symmetry. Chem. Sci. 2023, 15, 113–123. 10.1039/D3SC03928E. 38131074 PMC10732010

[ref44] Waldmann O. Relation between Electrostatic Charge Density and Spin Hamiltonian Models of Ligand Field in Lanthanide Complexes. Inorg. Chem. 2025, 64, 1365–1378. 10.1021/acs.inorgchem.4c04392. 39808919

[ref45] Alessandri R.; Zulfikri H.; Autschbach J.; Bolvin H. Crystal Field in Rare-Earth Complexes: From Electrostatics to Bonding. Chem.—Eur. J. 2018, 24, 5538–5550. 10.1002/chem.201705748. 29356203

[ref46] Briganti M.; Lucaccini E.; Chelazzi L.; Ciattini S.; Sorace L.; Sessoli R.; Totti F.; Perfetti M. Magnetic Anisotropy Trends along a Full 4f-Series: The fn+7 Effect. J. Am. Chem. Soc. 2021, 143, 8108–8115. 10.1021/jacs.1c02502. 34024105 PMC8297734

[ref47] Gil Y.; Aravena D. Understanding Single-Molecule Magnet properties of lanthanide complexes from 4f orbital splitting. Dalton Trans. 2024, 53, 2207–2217. 10.1039/d3dt04179d. 38193335

[ref48] Car P.-E.; Perfetti M.; Mannini M.; Favre A.; Caneschi A.; Sessoli R. Giant field dependence of the low temperature relaxation of the magnetization in a dysprosium(III)-DOTA complex. Chem. Commun. 2011, 47, 3751–3753. 10.1039/c0cc05850e. 21347457

[ref49] Cucinotta G.; Perfetti M.; Luzon J.; Etienne M.; Car P.-E.; Caneschi A.; Calvez G.; Bernot K.; Sessoli R. Magnetic anisotropy in a dysprosium/DOTA single-molecule magnet: beyond simple magneto-structural correlations. Angew. Chem., Int. Ed. 2012, 51, 1606–1610. 10.1002/anie.201107453. 22241599

[ref50] Briganti M.; Garcia G. F.; Jung J.; Sessoli R.; Le Guennic B.; Totti F. Covalency and magnetic anisotropy in lanthanide single molecule magnets: the DyDOTA archetype. Chem. Sci. 2019, 10, 7233–7245. 10.1039/C9SC01743G. 31588292 PMC6685353

[ref51] Jackson J. D. Classical Electrodynamics, third edition, international adaptation ed.; Wiley: Singapore, 2021.

[ref52] Messiah A. Quantum Mechanics, 11th ed.; North-Holland: Amsterdam etc., 1986; Vols. 1–2.

[ref53] Buckmaster H. A.; Chatterjee R.; Shing Y. H. The application of tensor operators in the analysis of EPR and ENDOR spectra. Phys. Status Solidi A 1972, 13, 9–50. 10.1002/pssa.2210130102.

[ref54] Duros O.; Juhin A.; Elnaggar H.; Chiuzbăian G. S.; Brouder C. General expressions for Stevens and Racah operator equivalents. J. Phys. A: Math. Theor. 2025, 58, 025207 10.1088/1751-8121/ad96fc.

[ref55] Görller-Walrand C.; Binnemans K. Rationalization of crystal-field parametrization, Handbook on the Physics and Chemistry of Rare Earths; Elsevier, 1996; Vol. 23, pp 121–283.

[ref56] Condon E. U.; Shortley G. H. The theory of atomic spectra, student edition ed.; Cambridge U.P: Cambridge, 2004.

[ref57] Sievers J. Asphericity of 4f-shells in their Hund’s rule ground states. Z. Phys. B - Condensed Matter 1982, 45, 289–296. 10.1007/BF01321865.

[ref58] Bethe H. Termaufspaltung in Kristallen. Ann. Phys. 1929, 395, 133–208. 10.1002/andp.19293950202.

[ref59] Schäffer C. E. The angular overlap model of the ligand field: theory and applications. Pure Appl. Chem. 1970, 24, 361–392. 10.1351/pac197024020361.

[ref60] Skomski R. Simple Models of Magnetism; Oxford graduate texts, Oxford graduate texts; Oxford University Press, UK: Oxford, 2008.

[ref61] Snyder J. P. Map projections: A working manual.

[ref62] Scheie A. PyCrystalField: software for calculation, analysis and fitting of crystal electric field Hamiltonians. J. Appl. Crystallogr. 2021, 54, 356–362. 10.1107/S160057672001554X.

