# Supplementary material for: Charge-Potential Model of Ligand Field in Lanthanide Complexes in the Single-Electron Space
Source: Inorg Chem. 2025 Apr 10;64(15):7666–81. doi: 10.1021/acs.inorgchem.5c00687 (PMC12015825; doi:10.1021/acs.inorgchem.5c00687)
Supplement: Supplementary file 1 — ic5c00687_si_001.pdf [file ic5c00687_si_001.pdf]

Supporting Information for

Charge-Potential Model of Ligand Field in Lanthanide Complexes in the Single-Electron Space

Oliver Waldmann

Physikalisches Institut, Universität Freiburg, 79104 Freiburg, Germany

Author email: oliver.waldmann@physik.uni-freiburg.de

Content

• Figure S1: Pictorial representation of the ground and excited states in the f orbital single-electron space for a strictly uniaxial ligand field. (page S2)

• Table S1: Table of a_{kq} factors for $k = 2,4,6$. (page S3)

• Table S2: Factors $K_{Mm}^{(J)(l)}$ for the trivalent lanthanide ions. (pages S4 - S8)

• Table S3: Coordinates and charges of the ligand atoms defining the ligand field potential $V_{DOTANa_3}(\mathbf{r})$. (page S9)

Figure S1

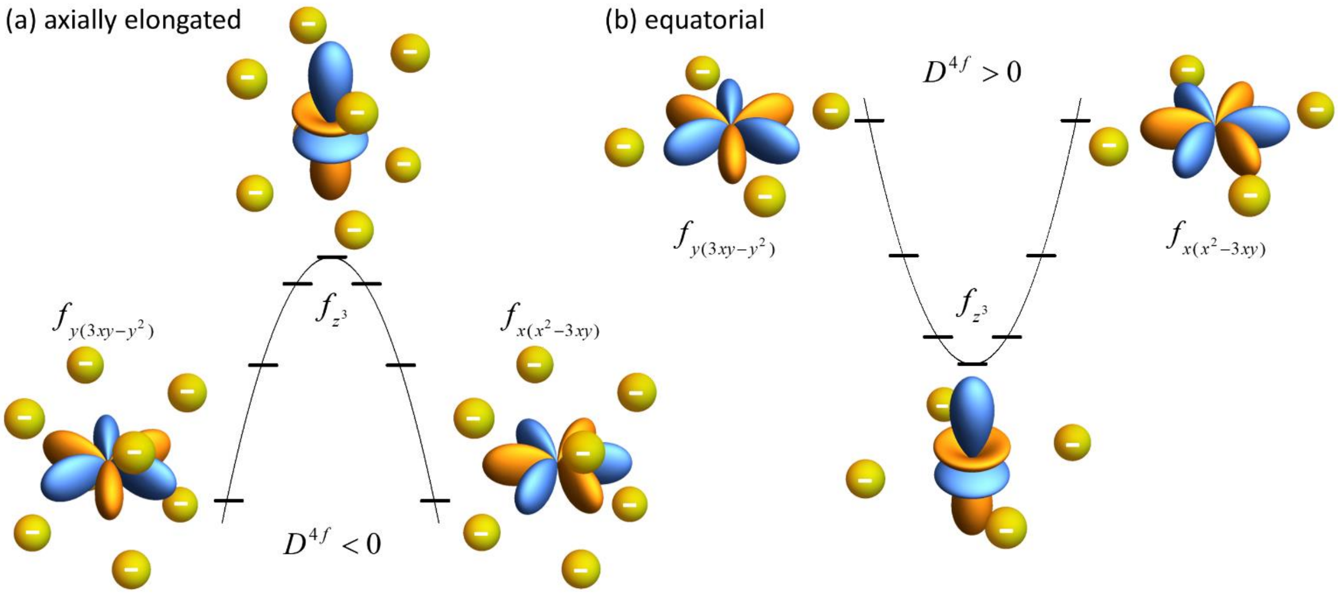

Figure S1: Pictorial representation of the ground and excited states in the f orbital single-electron space for **(a)** the axially elongated and **(b)** equatorial ligand field environments discussed in the text (only c_{20} is assumed to be non-zero). The negatively charged ligands are represented by yellow spheres, the f orbitals by spherical plots. In the axially elongated ligand field, the single-electron states $|3,\pm3\rangle$ or $f_{x(x^2-3xy)}$ and $f_{y(3xy-y^2)}$ orbitals, respectively, are lowest in energy since they minimize the Coulomb repulsion with the ligand charges, corresponding to a single-electron ligand field splitting parameter $D^{4f} > 0$. For the equatorial ligand field the situation is reversed and the single-electron state $|3,0\rangle$ or f_{z^3} orbital, respectively, minimizes the Coulomb repulsion, corresponding to $D^{4f} < 0$.

Table S1

Factors $a_{kq} = b_{kq}\sqrt{\frac{2k+1}{4\pi}}$ relating Stevens operators $\hat{O}_{kq}$ to tesseral harmonics Z_{kq} according to $Z_{kq} \equiv a_{kq}\hat{O}_{kq}$. In the table the values of b_{kq} are listed.

b_{kq}	$q=0$	$q=\pm1$	$q=\pm2$	$q=\pm3$	$q=\pm4$	$q=\pm5$	$q=\pm6$
$k=2$	$\frac{1}{2}$	$\sqrt{3}$	$\frac{1}{2}\sqrt{3}$				
$k=4$	$\frac{1}{8}$	$\frac{1}{4}\sqrt{10}$	$\frac{1}{4}\sqrt{5}$	$\frac{1}{4}\sqrt{70}$	$\frac{1}{8}\sqrt{35}$		
$k=6$	$\frac{1}{16}$	$\frac{1}{8}\sqrt{21}$	$\frac{1}{32}\sqrt{210}$	$\frac{1}{16}\sqrt{210}$	$\frac{3}{16}\sqrt{7}$	$\frac{3}{16}\sqrt{144}$	$\frac{1}{32}\sqrt{462}$

Note: Similar tables exist for relating the Stevens operators to the real Wybourne operators. The table entries differ however for $|q| > 0$ by a factor of $\sqrt{2}$ since the real Wybourne operators are defined without the factor $1/\sqrt{2}$ appearing in the definition of the tesseral harmonics Z_{kq}.

Table S2

Factors $K_{Mm}^{(J)(l)}$ for trivalent lanthanide ions. These factors are for calculating the energies ε_M of the $|JM\rangle$ states in the J multiplet space from the energies ε_m^{4f} of the $|lm\rangle$ states, or f orbitals, in the single-electron space, for the case of an uniaxial ligand field (see manuscript for details). The degeneracy of the f orbitals for $m = \pm 3, \pm 2, \pm 1$ is taken into account by doubling the respective values of $K_{Mm}^{(J)(l)}$. All values in the tables need to be divided by a common devisor, which is different for each lanthanide ion and given in the column "c.d." for the respective lanthanide ion.

For a $|JM\rangle$ level, the energy is calculated as

$$\varepsilon_M = 2K_{M,m=3}\varepsilon_3^{4f} + 2K_{M,m=2}\varepsilon_2^{4f} + 2K_{M,m=1}\varepsilon_1^{4f} + K_{M,m=0}\varepsilon_0^{4f}$$

where the values of $2K_{M,m=3}$, $2K_{M,m=2}$, $2K_{M,m=1}$, $K_{M,m=0}$ are given for each M in the rows of the tables.

The correctness of the values can be double-checked by the fact that the sum of the values in each row or in each column needs to be zero.

ion	*M*	*m* = 3	*m* = 2	*m* = 1	*m* = 0	c.d.
Ce^{III}	±5/2	4	-1	-2	-1	/7
	±3/2	-2	3	0	-1	
	±1/2	-2	-2	2	2	

ion	*M*	*m* = 3	*m* = 2	*m* = 1	*m* = 0	c.d.
Pr^{III}	±4	488	292	-464	-316	/1155
	±3	341	-506	229	-64	
	±2	-106	-74	-206	386	
	±1	-443	178	181	84	
	0	-560	220	520	-180	

ion	*M*	*m* = 3	*m* = 2	*m* = 1	*m* = 0	c.d.
Nd^{III}	±9/2	9417	8262	-3141	-14538	/66066
	±7/2	8647	-3638	-25051	20042	
	±5/2	3607	-19108	24859	-9358	
	±3/2	-6723	-3468	17049	-6858	
	±1/2	-14948	17952	-13716	10712	

ion	*M*	*m* = 3	*m* = 2	*m* = 1	*m* = 0	c.d.
Pm^{III}	±4	-9340	-7072	5332	11080	/66066
	±3	-7639	6732	15069	-14162	
	±2	-508	14416	-22516	8608	
	±1	10013	-5100	-4743	-170	
	0	14948	-17952	13716	-10712	

ion	*M*	*m* = 3	*m* = 2	*m* = 1	*m* = 0	c.d.
Sm^{III}	±5/2	-3341	-546	2223	1664	/9702
	±3/2	13	1638	-663	-988	
	±1/2	3328	-1092	-1560	-676	

ion	*M*	*m* = 3	*m* = 2	*m* = 1	*m* = 0	c.d.
Tb^{III}	±6	660	-264	-264	-132	/924
	±5	198	198	-264	-132	
	±4	-54	240	-54	-132	
	±3	-180	114	114	-48	
	±2	-236	-40	184	92	
	±1	-257	-152	191	218	
	0	-262	-192	186	268	

ion	*M*	*m* = 3	*m* = 2	*m* = 1	*m* = 0	c.d.
Dy^{III}	±15/2	1287	1287	-1716	-858	/3003
	±13/2	1287	-715	286	-858	
	±11/2	858	-1001	143	0	
	±9/2	264	-627	-297	660	
	±7/2	-330	-143	-319	792	
	±5/2	-828	227	109	492	
	±3/2	-1179	441	696	42	
	±1/2	-1359	531	1098	-270	

ion	M	$m = 3$	$m = 2$	$m = 1$	$m = 0$	c.d.
Ho^{III}	±8	2860	2860	2860	-8580	/20020
	±7	2860	2860	-12155	6435	
	±6	2860	-2145	-5148	4433	
	±5	2145	-5720	4576	-1001	
	±4	660	-5720	8932	-3872	
	±3	-1265	-2640	6930	-3025	
	±2	-3140	1735	1300	105	
	±1	-4490	5380	-4133	3243	
	0	-4980	6780	-6324	4524	

ion	M	$m = 3$	$m = 2$	$m = 1$	$m = 0$	c.d.
Er^{III}	±15/2	-715	-715	-715	2145	/5005
	±13/2	-715	-715	3289	-1859	
	±11/2	-715	715	1001	-1001	
	±9/2	-495	1595	-1639	539	
	±7/2	-55	1375	-2431	1111	
	±5/2	485	335	-1415	595	
	±3/2	965	-895	329	-399	
	±1/2	1245	-1695	1581	-1131	

ion	M	m = 3	m = 2	m = 1	m = 0	c.d.
Tm^{III}	±6	-1188	-1188	1584	792	/2772
	±5	-1188	1122	-726	792	
	±4	-558	912	114	-468	
	±3	198	198	576	-972	
	±2	828	-292	16	-552	
	±1	1227	-488	-901	162	
	0	1362	-528	-1326	492	

ion	M	m = 3	m = 2	m = 1	m = 0	c.d.
Yb^{III}	±7/2	-5	2	2	1	/7
	±5/2	1	-4	2	1	
	±3/2	2	0	-3	1	
	±1/2	2	2	-1	-3	

Table S3

Coordinates and charges of the ligand atoms used in the PCM-like ligand field potential $V_{DOTANa_3}(\mathbf{r})$ modeling the $[Dy^{III}DOTA(H_2O)Na_3]^{2+}$ molecule. The coordinates were determined using the Diamond software and the crystal structure file reported in P.-E. Car et al, Chemical Communications 2011, 47, 3751-3753, DOI https://doi.org/10.1039/C0CC05850E, moving the coordinate frame into the center of the Dy^{III} ion, and rotating the coordinate frame such that the *z* axis points into the direction of the oxygen atom of the apical H_2O molecule (O9 in the list below) and the *x* axis into the direction of the "middle" Na^+ cation (Na2 in the list below).

atom	*x* [Å]	*y* [Å]	*z* [Å]	charge [e]
Dy1	0	0	0	+3
O1	-0.420439	2.19385	0.647131	-0.5
O2	-2.19116	-0.27838	0.714703	-0.5
O3	1.57909	-4.02162	0.964046	-0.5
O4	3.97842	1.71283	0.82801	-0.5
O5	0.398602	-2.14482	0.825412	-0.5
O6	2.19809	0.404878	0.691167	-0.5
O7	-4.21209	-1.22994	0.612661	-0.5
O8	-1.91375	3.82482	0.920707	-0.5
O9	0	0	2.46566	0
Na1	-0.0677109	3.97003	2.44395	+1
Na2	3.82583	0	2.50153	+1
Na3	-0.0399117	-5.10585	2.20989	+1
